# Temperament & Character account for brain functional connectivity at rest: A diathesis-stress model of functional dysregulation in psychosis

**DOI:** 10.1038/s41380-023-02039-6

**Published:** 2023-04-04

**Authors:** Igor Zwir, Javier Arnedo, Alberto Mesa, Coral del Val, Gabriel A. de Erausquin, C. Robert Cloninger

**Affiliations:** 1 Washington University School of Medicine, Department of Psychiatry, St. Louis, MO USA; 2 University of Granada, Department of Computer Science, Granada, Spain; 3 University of Texas, Rio Grande Valley School of Medicine, Institute of Neuroscience, Harlingen, TX USA; 4 University of Texas, Long School of Medicine, Department of Neurology, San Antonio, TX USA; 5 Laboratory of Brain Development, Modulation and Repair, Glenn Biggs Institute of Alzheimer’s & Neurodegenerative Disorders, San Antonio, TX USA

**Keywords:** Neuroscience, Psychology, Predictive markers, Schizophrenia, Bipolar disorder

## Abstract

The human brain’s resting-state functional connectivity (rsFC) provides stable trait-like measures of differences in the perceptual, cognitive, emotional, and social functioning of individuals. The rsFC of the prefrontal cortex is hypothesized to mediate a person’s rational self-government, as is also measured by personality, so we tested whether its connectivity networks account for vulnerability to psychosis and related personality configurations. Young adults were recruited as outpatients or controls from the same communities around psychiatric clinics. Healthy controls (*n* = 30) and clinically stable outpatients with bipolar disorder (*n* = 35) or schizophrenia (*n* = 27) were diagnosed by structured interviews, and then were assessed with standardized protocols of the Human Connectome Project. Data-driven clustering identified five groups of patients with distinct patterns of rsFC regardless of diagnosis. These groups were distinguished by rsFC networks that regulate specific biopsychosocial aspects of psychosis: sensory hypersensitivity, negative emotional balance, impaired attentional control, avolition, and social mistrust. The rsFc group differences were validated by independent measures of white matter microstructure, personality, and clinical features not used to identify the subjects. We confirmed that each connectivity group was organized by differential collaborative interactions among six prefrontal and eight other automatically-coactivated networks. The temperament and character traits of the members of these groups strongly accounted for the differences in rsFC between groups, indicating that configurations of rsFC are internal representations of personality organization. These representations involve weakly self-regulated emotional drives of fear, irrational desire, and mistrust, which predispose to psychopathology. However, stable outpatients with different diagnoses (bipolar or schizophrenic psychoses) were highly similar in rsFC and personality. This supports a diathesis-stress model in which different complex adaptive systems regulate predisposition (which is similar in stable outpatients despite diagnosis) and stress-induced clinical dysfunction (which differs by diagnosis).

## Introduction

An outstanding feature of the human brain at rest is its spontaneous and organized functional activity, which accounts for most of the brain’s energy consumption [1–4]. Brain energy consumption varies little between rest and engagement in attention-demanding tasks, suggesting that brain function is not primarily reflexive or task-dependent [5–7]. Many questions about the brain’s intrinsic activity remain a mystery [8], but several lines of accumulating evidence suggest it is an internal representation of an individual’s personality that is continuously active in the background of their life. First, human personality is defined as the dynamic organization within the individual of the biopsychosocial systems by which the person both shapes and adapts uniquely to an ever-changing internal and external environment [9, 10], just as the connectome is dynamic, self-organized, adaptive, and idiographic [8]. Like the connectome [11], personality has collaborative components for emotional reactivity (i.e., temperament) and self-regulation (i.e., character) that are crucial for all aspects of health, including physical, mental, and social well-being [12–19].

Second, like personality [9, 10], the resting-state functional connectivity (rsFC) of healthy people is a large, sparse, complex network of interconnected modules with efficient small-world properties [20, 21]. In other words, there are multiple hubs with dense local connectivity, which may in turn be connected to other such hubs by longer-distance connections, so that a small number of connections between the hubs allows communication among all brain regions. Such pervasive connectivity is essential for the self-organized healthy functioning of living organisms, as found in the collaborative communication among the roots of trees [20, 22], in the neural networks of animals [23], and the social networks of human communities [23, 24].

Third, reduced connectivity among hubs results in inefficient and dysfunctional integration of the brain, as in patients with schizophrenic or bipolar psychoses [25–29] and related conditions such as personality disorders with schizotypal [30–33], borderline [34, 35], narcissistic [36], or neurotic features [37]. Most studies of rsFC in individuals with psychoses and related conditions show prominent loss of prefrontal cortical connectivity, including connectivity within the prefrontal cortex and its connections with parietal, temporal, limbic, and cerebellar hubs, compared to healthy individuals [25, 38].

Fourth, the decreased rsFC in patients with psychoses leads to decreases in modularity and efficiency of integration (i.e., loss of small world properties). In cognitive and emotional terms, reduced rsFC in psychosis is associated with impaired insight and judgment because of distorted perceptions and unrealistic representation and execution of new forms of organized goal-directed behavior, which is widely accepted as the basic function of the prefrontal cortex in collaboration with other brain regions [39].

However, many aspects of the relations between rsFC and human brain functions remain unclear, particularly the differences between psychoses and rational creative states [40]. Psychotic and psychotic-prone individuals may show increased efficiency and connectivity between some hubs, so that connectivity is not uniformly reduced in dysfunctional configurations of rsFC. In particular, the rsFC of the Default Mode Network (DMN) is abnormally increased in most studies of schizophrenia and schizotypy [25, 30, 38], as well as in creatively divergent thinking in healthy prefrontal network configurations [41, 42]. Furthermore, the extent to which individual networks show both increased and decreased connectivity in different subjects with psychoses and related conditions is likely to have been underestimated because most studies have relied on average connectivity in samples [27–29, 43].

### Relating human brain functions to rsFC

The Human Connectome is a promising intermediate phenotype that functions between phenomics (including clinical psychopathology, personality, and behavior) and more basic omics (including genomics, transcriptomics, and proteomics). In studies of individual differences, investigators often seek to relate individual differences in functional connectivity to specific differences in structural connectivity, personality, and clinical psychopathology [44, 45]. However, simple linear correspondence between individual networks and behaviors is unlikely because it neglects the complex connectivity of the whole brain. The trait-like features of complex adaptive systems are relatively resilient configurations that can abruptly change in response to small shifts in conditions at tipping points: that is, as shown in longitudinal studies of human personality [10], they are “meta-stable”, not fixed traits with discrete boundaries.

The brain is organized as several networks with distinct but collaborative roles [46–49]. Their circuitry overlaps partially, which facilitates functional interactions [50]. The complexity and heterogeneity of rsFC have made it difficult to characterize the functions of individual networks precisely because multiple networks operate collaboratively and the correlations among networks vary between individuals [47] and develop across the lifespan in relation to genetic and environmental influences [51, 52] and individual experiences [53, 54]. Average values of connectivity in heterogeneous groups with the same clinical diagnosis may say little about what is happening in a particular individual.

Nevertheless, these challenges apply to all complex phenomena, and methods appropriate for characterizing complex systems are available. In particular, recent work with personality and schizophrenia has shown that it is possible to identify subgroups of individuals with distinctive genotypic, environmental, and phenotypic characteristics [14]. The phenotypic characteristics include clinical signs and symptoms [55], temperament and character domains of personality [56–58], learning networks [12], and neuroimaging characteristics [59]. Rather than focusing on individual diagnoses or traits, we have found that the complex traits are most appropriately measured as multi-dimensional configurations that correspond to the higher-order organization of the complex system [60].

For example, personality traits measured by the Temperament and Character Inventory (TCI) are complex in their genetic and environmental antecedents as well as in cognitive and emotional features that influence health [9, 61]. Different TCI trait configurations provide specific measures of schizotypal personality disorder and susceptibility to psychosis [16, 62, 63], cyclothymia, neuroticism and susceptibility to mood disorders [64, 65], and learning by behavioral conditioning, intentional self-control, and self-awareness [12, 13]. Such configural analysis allows deconstruction of a complex system into its components as well as the analysis of integrative interactions among components, as we did for human personality [12, 13, 58].

In view of the availability of methods to understand complex systems, the brain’s rsFC is a particularly promising target for multiple reasons. First, the intrinsic activity of the resting human brain accounts for most of its energy consumption, which varies less than 5% between rest and attention-demanding tasks [5–7]. Brain rsFC networks are highly stable across multiple scans and imaging sessions over months or years [8]. The FC networks at rest across sessions are highly stable in the same individual, correlating at *r* = 0.8 to 0.9 [8, 66]. The patterns are relatively stable across tasks (*r* = 0.5 to 0.9), but even small changes in FC may produce a wide variety of cognitive and behavioral states, as expected for complex adaptive systems [8].

Second, the organization of rsFC corresponds generally to brain structural connections, particularly the positive correlations among regions [67], which provides a potential basis for validation of groups of people with heterogeneous configurations of rsFC with meta-stable (trait-like) properties [68]. Direct intracranial brain stimulation during neurosurgery documents distinct temporal and directional patterns of signal flow within and between rsFC networks in the human brain [69–71]. The positive correlations among regions remain stable even under general anesthesia, suggesting the positive rsFC reflects structural connectivity independent of being awake and subjectively aware, whereas the negative correlations also observed in conscious subjects are nearly all lost under general anesthesia [67]. However, this does not mean that positive functional connectivity depends on structural connectivity: in fact, frequent positive functional connectivity often leads to later structural connectivity. For example, inputs to dendrites that fire together tend to wire together, forming synaptic clusters within individual branches of dendritic networks that carry out dynamic non-linear computations [72, 73].

Third, rsFC is predictive of interindividual differences in a wide range of phenotypes [74, 75], including personality configurations associated with vulnerability to psychosis [30–37]. Like rsFC, self-reported measures of temperament and character are moderately to strongly stable over time regardless of a person’s health status (~0.7 to 0.9) [62, 76–78].

Fourth, rsFC can flexibly reconfigure across a range of cognitive and behavioral states and tasks in adaptive responses to changing conditions and experiences, including psychosocial development [53, 54], adverse events [79, 80], and therapy [8, 81, 82]. Their reconfiguration can be trained, so they show promise as biomarkers to plan precise individualized interventions [8, 83–85].

### Potential Confounding Variables

Despite these promising features of rsFC as an intermediate phenotype between clinical phenomics and omics, there have been challenges in clinical applications when individual laboratories did small studies with different protocols in early efforts to reduce motion artifacts and to enhance signal to noise ratios [7, 86, 87]. Fortunately, standardized protocols have now been developed that can generate replicable results at multiple sites for the Human Connectome Project [88], as we used in the current study. Nevertheless, several factors in subject selection can led to inconsistent results in both structural and functional connectivity studies. Prior studies of white matter microstructure have usually found abnormalities in long-range association tracts when subjects with schizophrenia (SZ) or bipolar disorder (BP) are compared to healthy controls by fractional anisotropy (FA), a technique to measure asymmetric diffusion of water molecules along neuronal white matter tracts [59, 89]. This index of neuronal integrity is thought to reflect differences in the myelination and organization of white matter tracts, which also naturally depend on the developmental factors, such as age, gender, and health of subjects. Consequently, it is not surprising that findings about FA have varied in the strength of differences and the regions affected across studies because of a variety of influences that are naturally confounded with psychiatric diagnosis and related personality profiles. Divergent findings across studies of white matter microstructure are associated with a variety of factors, including age, gender, physical health, brain injury, clinical state, duration of illness, and the medications used in treatment [89].

Likewise resting-state functional connectivity based on correlations in spontaneous fluctuations in blood-oxygen- level-dependent (BOLD) signals in different brain regions are often associated with variability of demographic [54, 90, 91], health [90, 91], and medication history [92–94]. Fortunately, the effects of demographic variables and physical health are minimal within groups of young adults [90, 91], as selected in this study.

It is noteworthy that the subject variables that can confound studies of structural and functional connectivity are also associated with differences in the natural histories of different clinical disorders. For example, men with schizophrenia more often present with onset of psychosis earlier than do women [95], and people with schizophrenia are more often treated with neuroleptics than are those with bipolar disorder [89]. Therefore, the variable findings about brain connectivity in association with demographic, clinical, and treatment factors may be partly explained by the existence of subgroups of patients with distinct patterns of rsFC within heterogeneous diagnostic groups, such as groups including both BP and SZ patients. That is, differences in rsFC may occur largely within transdiagnostic groups. Such transdiagnostic groups of patients are also suggested by the occurrence of overlapping features of SZ and BP in the same individual, as in schizoaffective disorders, and in the same family [96].

### Goals of analysis

In this report we will deconstruct the dysfunctional integration of the brain’s rsFC to uncover groups of young adults with distinct patterns of rsFC within and across traditional diagnostic groups using standardized protocols from the Human Connectome Project. We will test the validity of any differences among transdiagnostic rsFC groups by independent measures of structural connectivity, personality, and clinical signs and symptoms not used to identify the transdiagnostic rsFC groups. We will also test for association of any of the identified rsFC groups with a specific diagnosis or potentially confounding demographic, health, or treatment effects.

Specifically, we will test how the personality and clinical characteristics observed in subjects correspond to the functions attributed to the canonical networks of the Human Connectome Project that are expected to provide a model of self-governance.

### Hypotheses to be tested

To formulate our hypotheses, we observed that, in healthy individuals, the human prefrontal cortex plays a crucial role in the development of insight and judgment and related neuroadaptive processes for regulating and integrating a person’s habits, goals, and values through its connections with other brain regions [13, 83, 84, 97, 98]. In contrast, we observed that, in individuals with psychoses or at high risk for psychosis due to personality disorders, the brain’s rsFC shows prominent disruption of prefrontal cortical connectivity [25, 38, 99].

Six distinct rsFC networks involving the human prefrontal cortex are proposed to mediate self-regulatory cognitive and emotional functioning in a collaborative manner [11, 100]. These include four networks with putative self-regulatory (top-down) functions: self-awareness and internally directed evaluation (Default Mode - DMN), intentional self-control (Cingulo-Opercular-CON, Fronto-Parietal-FPN), and persistent goal-directed attention (Dorsal Attention-DAN). Two other prefrontal networks regulate the plasticity of involuntary attention and orienting in response to bottom-up emotionally aversive or unfamiliar stimuli (Salience-SN) and novel or unexpected stimuli (Ventral Attention-VAN) across sensory modalities [46–50]. Other brain networks are presumed to be automatically co-activated with the prefrontal networks in different collaborative patterns that depend on the configuration of the prefrontal networks as an internal representation of self-governance of the person [101, 102].

We hypothesized that different interaction patterns of the prefrontal cognitive-emotional networks and related automatically-coactivated networks would be distinguished from one another in their structural connectivity, personality configurations, and clinical characteristics. Specifically, we hypothesized that these six prefrontal networks interact with one another in ways that influence a person’s self-regulation and risk of psychosis. According to prior work, when a person functions in a way that promotes health, their self-governance is rational, which includes health-promoting executive functions (i.e., self-directedness, including purposeful goal-seeking, resourceful problem-solving, responsible preparatory-planning, flexible multi-tasking), legislative functions (i.e., cooperativeness, including social tolerance, helpfulness, empathy, and fairness), and judicial functions (i.e., self-transcendence, including creative insight, coherent fluidity of thought, oceanic feelings, and altruistic values) [9, 103].

When such rational self-governance is sustained (i.e., maintained persistently despite intermittent reinforcement), emotional reactivity, attachments, and habits are self-conditioned to be reliably in accord with a person’s goals and values [18, 104, 105]. Therefore, we hypothesized that when irrational emotional drives overwhelm self-regulation, as in psychosis, self-government is disrupted by low self-directedness (i.e., aimless, helpless, inflexible, irresponsibly blaming others and external circumstances), low cooperativeness (i.e., prejudiced, hostile, self-centered, and opportunistic), and unrealistic insight and judgment (i.e., magical ideation, incoherent flow of thought, feelings of separation, and self-serving values).

More specifically, we hypothesized that the four top-down rsFC prefrontal networks (DMN, CON, FPN, and DAN) are primarily involved in cognitive processes of self-aware consciousness and intentional self-control associated with individual differences in the configuration of human character [12, 13, 56], as in personality configurations with low scores in TCI Self-directedness, Cooperativeness, and Self-Transcendence (sct, as in apathetic characters), low scores in Self-directedness and Cooperativeness (scT, as in schizotypal characters), and low scores in only Self-directedness (sCT, as in cyclothymic characters). In contrast, we hypothesized that the SN and VAN are involved in bottom-up involuntary sensory and emotional reactivity and, in conjunction with the automatic cortico-striato-cortical loop, with regulation of habits that are associated with individual differences in human temperament [13, 57].

We further hypothesized that TCI character profiles of apathetic, schizotypal, and cyclothymic personality may be associated with schizophrenic and/or bipolar psychoses, but that the schizotypal character will be more frequent in schizophrenic psychoses whereas apathetic and cyclothymic characters will be more frequent in bipolar psychoses. We expected that the self-regulatory dysfunction associated with psychoses may result from either overactivity or underactivity of integrated functional configurations of the four top-down rsFC prefrontal networks (DMN, CON, FPN, and DAN) [28]. In contrast, we hypothesized that bottom-up prefrontal networks regulating emotional reactivity (VAN, SN) were related to the involuntary emotional drives measured by TCI temperament dimensions, as supported by prior research [62, 104–110].

The personalities of healthy adults are characterized by nearly equal proportions of people with organized characters (SCt) or creative characters (SCT) combined with temperament configurations that are reliably in accord with rational goals and values (i.e., characterized by high ratings on Persistence (P) and Reward Dependence (R) and low ratings on harm avoidance (h) and novelty seeking (n)) [12]. The strong self-regulatory functioning of their self-directed and cooperative characters dominates and shapes their habits to be in accord with their rational goals and prosocial values [12, 13, 18, 111]. In contrast, the personalities of unhealthy adults, particularly those with or at risk for psychosis, have strong emotional drives opposite to reliable temperaments (namely, H, N, r, p) that dominate the weak and unrealistic self-regulatory functioning typical of people with apathetic (sct), schizotypal (scT), or cyclothymic characters (sCT) [12, 62, 63, 112, 113]. Consequently, if psychosis results from dysfunctional variation in the same rsFC networks found in healthy individuals (rather than secondary to unique and discrete pathogenic traits [114, 115]), we hypothesized that in psychoses, strong emotional reactivity (as measured by temperament and/or by rsFC of bottom-up prefrontal networks) would dominate the weak and dysfunctional self-regulation of psychotic individuals (as measured by character and/or by rsFC of top-down prefrontal networks).

## Subjects and Methods

### Subjects

All study protocols and recruitment procedures were approved by the Institutional Review Board of Washington University Medical School (WUMS) in St. Louis, MO. All participants (both outpatients and controls) were recruited from the communities around psychiatric clinics affiliated with WUMS in two ways: first, advertisements using posters and flyers soliciting young adults aged 18 to 30 years in psychiatric clinics and the public spaces of surrounding communities; second, direct contact through the WUMS research recruitment service (“Volunteers for Health”), which maintains a registry of people with a range of disorders and healthy adults interested in research. All subjects gave written informed consent prior to participation.

All subjects were screened for inclusion or exclusion criteria and diagnosed based on a consensus between a research psychiatrist and a trained research assistant who used the Structured Clinical Interview for DSM-IV Axis I Disorders (SCID-I). Selected subjects who satisfied pre-established inclusion and exclusion criteria included groups of 30 healthy controls and 62 patients with bipolar disorder (BP; *n* = 35) or schizophrenia (SZ; *n* = 27). Control subjects were required to have no lifetime history of psychotic or mood disorders. Subjects with BP or SZ were required to be clinically stable outpatients. In addition, to minimize clinical heterogeneity within the BPD group, only participants with a history of euphoric mania (versus mania characterized by primarily irritable mood) were included in the study.

Participants were excluded if they: (a) met DSM-IV criteria for substance dependence or severe/moderate abuse during the prior 6 months; or (b) had a clinically unstable or severe general medical disorder; or (c) had a history of head injury with documented loss of consciousness or neurological sequelae.

### Clinical Assessment

Psychopathology was assessed by a trained Masters-level research assistant using the Scale for the Assessment of Negative Symptoms (SANS) and the Scale for the Assessment of Positive Symptoms (SAPS) [116]. Specific subscale scores were summed to derive measures of positive symptoms (i.e., hallucination and delusion subscales), disorganization (i.e., positive formal thought disorder, bizarre behavior, and attention subscales), and negative symptoms (i.e., flat affect, alogia, anhedonia, avolition, and asocial subscales). Estimates of chronic psychotic and affective symptoms were derived using the Washington Early Recognition Center Affectivity and Psychosis Screen (WERCAP) [117], a self-assessment tool. Personality traits were obtained using the online version of the Temperament and Character Inventory-Revised (TCI-R) with 140 items rated on a five-point Likert scale [118]. Correspondence of TCI profiles to DSM-5 personality disorders is noted throughout this article [118], and in relation to other psychometric tests in Supplementary Information describing personality correlates of the rsFC groups, and elsewhere in detail for psychiatric disorders and other psychometric tests [14, 119].

### Image Acquisition

Scans were run using a 32-channel head coil on a customized Siemens 3T “Connectom” MRI scanner, which was previously used for collecting the Human Connectome Project – Young Adult (HCP-YA) data and housed at Washington University in St. Louis [88]. The scanning protocol used identical parameters for individual scans as that of the HCP-YA, which is extensively described in the literature [88, 120, 121]. However, the overall structure of the HCP-YA protocol was consolidated to 3 total imaging sessions (rather than 4) by: (i) only acquiring a single T1w and T2w scan (rather than two of each) and (ii) only acquiring 3 of the 7 fMRI task scans collected by HCP-YA. The 3 sessions were typically collected over a period of two days.

Briefly, T1-weighted MPRAGE images were acquired at 0.7 ×0.7 ×0.7 mm resolution. Four 15-minute resting-state BOLD runs were acquired at 2 × 2 x 2 mm resolution, with images collected every 0.7 s; the two runs (one each of left-right/right-left phase-encoding) with minimal in-scan motion were selected for each participant. Resting-state scans were acquired using a T2*-weighted multiband of 8 echo-planar imaging sequence with 72 axial slices per volume, field of view of 208 mm, echo time of 33.1 ms, repetition time of 720 ms, and flip angle of 52°. None of the task fMRI data are analyzed in the present report.

Diffusion image acquisition has been previously described [89]. Briefly, a full dMRI session included 6 runs (each approximately 9 minutes and 50 seconds), representing 3 different gradient tables, with each table acquired once with right-to-left and left-to-right phase encoding polarities, respectively.

### Functional and structural connectivity image processing

Diffusion tensor imaging (DTI) scans and analysis based on Tract Based Spatial Statistics (TBSS) was performed as described elsewhere [122].

Functional MRI data were run through minimal preprocessing pipelines, as previously reported [121] (see Supplementary Information).

Functional network nodes were determined using the 264-ROI atlas defined by Power et al. [123], and 36 subcortical ROIs from the Brainnetome atlas [124]. Each node was assigned membership to its most likely 12 Power atlas-based functional networks (auditory, cingulo-opercular, context, default mode, dorsal attention, frontoparietal, perception, somatomotor-face, somatomotor-body, salience, ventral attention, and visual) and 4 Brainnetome atlas-based networks (amygdala, entorhinal-hippocampus, striatum, and thalamus). For each node, 6 mm spherical ROIs were used. For the convenience of a broad readership, a descriptive summary of these 16 networks and their known functions is provided in Supplementary Information (see Notes on Connectivity Circuitry). Components of each canonical network’s functional and structural connectivity is provided in Supplementary Table S1.

BOLD time courses for each node were computed by averaging timeseries for all voxels within each node. These average timeseries were then correlated, resulting in a 369×369 whole brain correlation matrix.

### Computational analysis of connectivity networks

**Fig. 1:**
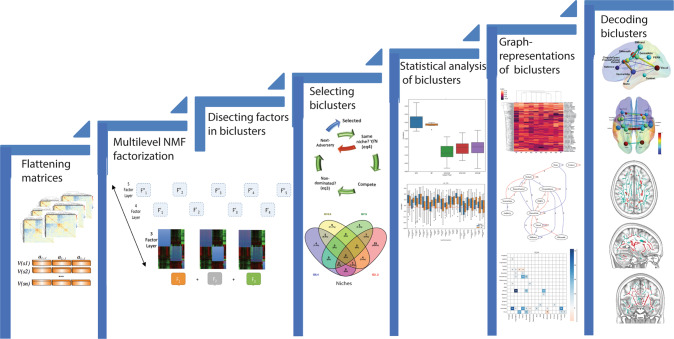
Specific description of method for group identification. Groups were uncovered in six steps, as detailed in Supplemental Information: (1) Preprocessing datasets by flattening matrices (Fig. S6); (2) Identifying optimal functional connectivity sets by multilevel NMF factorization: (2.1) Mathematical description of the NMF (Figs. S5-S7); (2.2) Decomposing the data into a multilevel family of sub-matrices (Fig. S7); (3) Dissecting factors in biclusters, including (3.1) Dissecting NMF k factors into sub-matrices or biclusters which are interpreted as fMRI sets (Fig. S7), and (3.2) Learning the W and H matrices of FNMF; (4) Selecting biclusters: Multi-view and optimally assembling the families of sub-matrices (Fig. S10); (5) Statistical analysis of biclusters (Figs. S2, S9); (6) Graph and matrix representations of biclusters (Fig. S8), including (6.1) Displaying biclusters extracted after factorization (Figs. S2, S9), (6.2) Displaying and decoding TBSS biclusters and transforming them back to native space (Fig. 2, S1, S6), and (6.3) Displaying and decoding fMRI biclusters and transforming them back to native space (Fig. 3, S3, S6).

Diffusion tensor imaging (DTI) analysis based on Non-negative Matrix Factorization (NMF) of neuroimages was performed as described elsewhere [122] and available at the NMF-based DTI-TBSS Analysis (NDTA web server (http://picu.ugr.es/ndta/). This method was extended in this project to provide customized functional connectivity analysis (see Supplemental Information). We sought to identify naturally occurring and homogeneous groups of subjects sharing distinct patterns of organization of brain functional connectivity in their resting-state fMRI data by using a data-driven approach based on machine learning and optimization research techniques [125–129], as described in our previously published articles using the same approach [12, 56–59]. We used multi-level NMF to identify these groups in an unsupervised fashion. In other words, we identified naturally occurring groups of subjects without restrictive assumptions about the number of the groups or the patterns of their distinguishing features. The groups were identified using only rsFC data regardless of their diagnosis, personality, other clinical features, or structural connectivity. Specifically, the level of rsFC for each network in each of the 62 patients was rated as higher or lower than the mean rsFC for that network in 30 healthy controls, and then NMF was carried out to identify groups within the 62 patients who had significantly different patterns of correlations among their networks and those of the healthy controls. The steps involved in using NMF and related procedures to identify and describe the patterns of high and low rsFC that specify the groups are summarized in Fig. 1 and detailed in Supplementary Information.

Regarding the statistical power of the sample, it can be readily observed that the 62 patients provided about 10 subjects per independent variable to test hypotheses about the 6 prefrontal rsFC networks or 7 personality variables, which satisfies a common rule of thumb for our planned analyses. More specifically, the sample size of 62 patients and 30 controls was originally estimated to give the power to detect true differences between patients and controls with an effect size of 0.2 or greater with a probability of 80% or more and a risk of false positives of 5% after adjustment for multiple comparisons (statistical toolbox of Matlab r2022b). In practice, we had substantially greater power than originally estimated because the number of comparisons was reduced by our use of a small number of groups identified by NMF instead of 92 individuals.

The Fuzzy NMF machine learning method identifies matrices composed of subgroups of subjects with distinct features, which in this case are correlations between rsFC networks [55, 130–132]. These matrices have different sizes because all features and/or subjects are not forced to belong to each matrix. Moreover, these matrices are defined at different levels of granularity to provide a multifaceted description of all possible groups, ranging from specific groups characterized by many shared features in a few subjects to broad groups with many subjects sharing a few features [133, 134]. It is expected that the constituent subjects and/or features can participate in more than one matrix because rsFC networks operate collaboratively and the matrices encode these collaborative relationships. That is, correlated groups necessarily share subjects and/or features. Therefore, no restrictive assumptions were made about the extent of separation of groups or their number.

The identified rsFC groups are called biclusters when represented by matrices because each group is a relationship specified by both its subjects and their distinguishing features. In the terminology of graph theory, biclusters are bipartite graphs with cliques (Supplementary Information). Groups were selected to maximize jointly their specificity, sensitivity, and the multifaceted coverage of subjects and features by using optimization techniques from the operations research field [125–129] (see also Supplementary Information; algorithm code available upon request).

All subjects, regardless of diagnosis or control status, are included in the analysis, so it was desirable for us to have roughly equal numbers of subjects who were healthy, patients with schizophrenia, or patients with bipolar disorder. However, the identification of groups is exploratory and requires validation with independent data. Therefore, we next evaluated the individuals within each identified rsFC group by their shared features in other domains that had not been used to identify them, which included structural microstructure using FA, personality profiles, and other clinical characteristics. Since none of the data used for the second step of the analysis were part of the clustering algorithm, group differences cannot be attributed to over-fitting or other sources of bias; rather, they provide an independent test to validate the identified functional connectivity groups.

## General statistical analysis

Statistical significance was assessed by comparing the connections characterizing each group (bicluster) as measured by the correlation coefficient with the same connectivity values of subjects not involved in that group, including comparisons with controls and/or subjects in other groups, using one-way ANOVA and pairwise t-tests (R version 2.15.1) and applying Bonferroni correction (Supplementary Tables S1–S3, Figures S1, S2)). Significance in each domain of knowledge, including structural connectivity, personality, and clinical data was calculated in the same fashion (Supplementary Tables S4–S7, Figures S3–S5). Significance testing of demographic and other potential confounding variables also used ANOVA and pairwise t-tests, as well as pairwise Chi-squared tests, and the effect size of intergroup differences as the correlation coefficient using R version 2.1. These procedures properly account for differences in group size and any overlap in group membership.

The explanatory power of personality features to account for membership in rsFC groups was calculated as a set of stepwise logistic regressions to predict membership in each rsFC group versus all other patients. That is, the seven personality variables of the 62 patients, who had independently been divided into subgroups based on rsFC alone, were used to estimate how well they distinguished members of each group from all the others. Then the regression between actual group membership and the regression values provided an estimate of the explained variance for each group after adjusting for multiple tests, including the squared regression (R2), the F-statistic and its probability p, and the root mean square error (RMSE), using the statistical toolbox of Matlab r2022b.

## Results

### Identification of functional connectivity groups

Our search strategy uncovered five groups of patients with distinct functional connectivity patterns. The rsFC pattern in each group differed from the other groups and from healthy controls, along with associated clinical features related to our hypotheses (Table 1, Supplementary Tables S1–S6). In Table 1, the different patterns in each group are shown by describing rsFC in each network as positive when significantly greater than in healthy controls and as negative when significantly less than in healthy controls. As predicted, the five groups identified within the 62 patients were distinguished primarily by distinct patterns of the four top-down (self-regulatory) prefrontal networks (DMN, CON, FPN, DAN) and two bottom-up prefrontal networks that react to aversive or novel external stimuli (i.e., SN and VAN). Eight other networks were co-activated with the prefrontal networks (Table 1).

**Table 1:** Distinct organization of the resting-state Functional Connectivity of five groups of subjects and their associated temperament, character, and clinical features.

Functional Type (clinical)	rsFC Network	Group 1 Avoidant-Anhedonic	Group 2 Sensitive- Disorganized	Group 3 Asocial- Blocked	Group 4 Fragile-Avolitional	Group 5 Explosive -Inattentive
**PFC top-down**	Default Mode	**Positive**	**Positive**	**Negative**	Negative	**Negative**
	Cingulo-Opercular	Positive	**Positive**	**Positive**	**Negative**	**Negative**
	Fronto-Parietal	**Positive**	**Positive**	**Positive**	Positive	Positive
	Dorsal Attention	Positive	**Positive**	**Positive**	Positive	**Negative**
self-regulatory functioning/ character		Defensive Control (s)	Overattentive Schizotype (scT)	Hostility Mistrust (cT)	Avolition Apathy (s)	Inattentive Schizotype (scT)
**PFC Bottom-up**	Ventral Attention	**Positive**	**Positive**	**Negative**	Negative	Negative
	Salience	--	Positive	--	--	--
emotional reactivity/ temperament		Harm Avoidance (H)	Ambivalence (HN)	Novelty Seeking (N)	Fragility (pHN)	Explosive Borderline (rHN)
**Automated** **modules**	Visual	Positive	**Positive**	**Negative**	**Negative**	**Negative**
	Auditory	--	Positive	**Positive**	**Negative**	--
	Thalamus	--	--	Positive	Negative	--
	Striatum	--	Positive	Positive	**Negative**	--
	Context	--	--	**Positive**	--	Negative
	Perception	--	Positive	Positive	Positive	Negative
	SM hand	--	**Positive**	**Positive**	N**egative**	**Negative**
	SM mouth	--	**Positive**	Negative	**Negative**	Positive
	Amygdala	--	**--**	--	--	--
	Entorhinal	--	**--**	--	--	--
(Negative sx)		Anhedonia Asociality	Alogia Blunt Affect	Blocking	Avolition Apathy	Inattention
(Positive sx)		--	Bizarre behavior Thought disorder	Somatic Delusions	Hallucinate Delusions	--

rsFC in patients compared to healthy control is shown as positive when significantly greater than in healthy controls, negative when significantly less than in healthy controls. Differences that are bold are highly significant. Other observations are not significantly different (--). Significantly associated traits in each group are TCI character traits of low self-directedness (s) and cooperativeness (c), high/low Self-Transcendence (T/t), and TCI temperament traits of low persistence (p) and reward dependence (r), and high Harm Avoidance (H) and Novelty Seeking (N). Positive/negative symptoms are significant SAPS/SANS ratings.

**Fig. 2:**
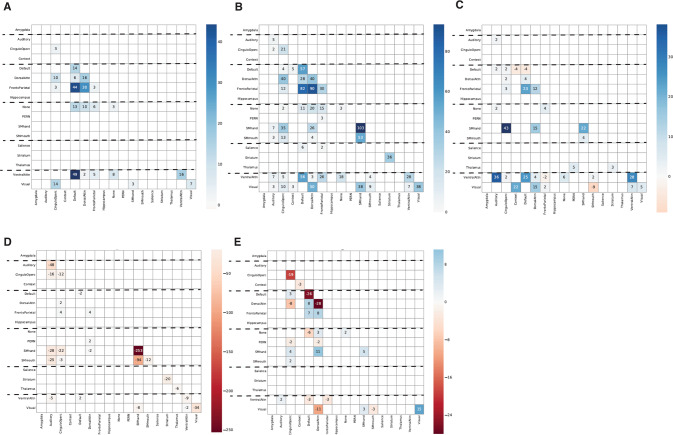
Matrix representation of the five fMRI connectivity groups that account for 97% of the connectivity in SZ and BP patients. Number of connections and their relative importance are shown and color coded [blue indicates positive connections (correlation greater than in controls) whereas red shows negative connections (correlation less than in controls)]. The number in the cells represents edges (i.e., links) are the functional connections between nodes (i.e., component brain regions or fMRI networks) that specify the connectivity networks (see also Figures S2 and S9). **A**–**E** represent each of the five sets that describe different patterns of connections among the nodes: **A** the Avoidant-Anhedonic (group 1), **B** the Sensitive-Disorganized subjects (group 2), **C** the Asocial-Blocked subjects (group 3), **D** the Fragile-Avolitional subjects (group 4), and **E** the Explosive-Inattentive individuals (group 5).

The distinct pattern of functional connections among all 16 networks and regions are depicted for each group of patients as a bicluster matrix in Fig. 2. Both the number of connections and their strength relative to controls among each pair of regions are shown within the matrix of Fig. 2.

Information about the number of subjects, connections, and diagnoses of subjects in each connectivity group is summarized in Table 2. Individuals in each connectivity group were all diagnosed with SZ or BP (risk = 1) and accounted for 97% of the total number of subjects. The proportions of cases of SZ and of BP were nearly the same in each connectivity group. We had expected only a few healthy controls to be included in rsFC groups with the patients because connectivity strength was evaluated in relation to the mean of the controls (i.e., higher, or lower), but in fact no subject in the connectivity groups was a healthy control. The significance of the differences among the group means was tested by ANOVA for patients versus controls (Table 2).

**Table 2:** Functional biclusters (coverage of 97%). Five sets selected based on subject’s coverage, risk, and difference from “others” (uniqueness of subjects and/or connections).

Network (Name)	ANOVA p	Connections #	Subjects #	SZ #	BP #	Controls #	Risk	SZ %	BP %
Group 1 (Avoidant-Anhedonic)	1.2 E−07	279	11	5	6	0	1.00	45	55
Group 2 (Sensitive-Disorganized)	9.9 E−12	1077	27	10	17	0	1.00	37	63
Group 3 (Asocial-Blocked)	6.1 E−06	366	10	4	6	0	1.00	40	60
Group 4 (Fragile-Avolitional)	6.5 E−15	627	35	17	18	0	1.00	49	51
Group 5 (Explosive-Inattentive)	9.3 E−04	209	16	8	8	0	1.00	50	50

Individuals in each fMRI groups were all diagnosed with SZ or BP (risk = 1) and accounted for 97% of the connections observed. None were controls. Significance was tested by ANOVA for patients versus controls.

**Fig. 3:**
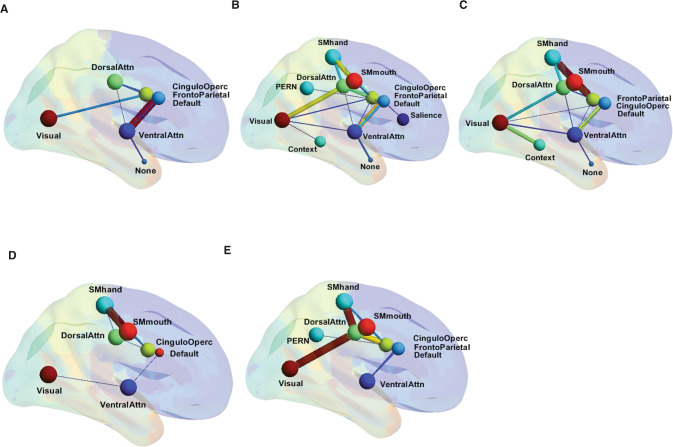
Graph representation of the five fMRI connectivity groups using BrainNet Viewer. Thicker edges correspond to stronger and higher number of correlations (only the most notorious edges are shown). **A**–**E** represent each of the five sets that describe different patterns of connections among the nodes: **A** The Avoidant-Anhedonic (group 1), **B** the Sensitive-Disorganized subjects (group 2), **C** the Asocial-Blocked subjects (group 3), **D** the Fragile-Avolitional subjects (group 4), and **E** the Explosive-Inattentive individuals (group 5).

The distinctive functional connectivity patterns of each of the groups we uncovered can be depicted as bicluster matrices (as in Fig. 2) or as graphs of brain connectivity (as in Fig. 3). The connectivity can be quantified in terms of the number and strength of their connections (i.e., by the number of pairs of regions with significantly correlated BOLD signals as well as by the strength of those correlations, as depicted in Figs. 2 and 3). Members of each functional connectivity group differ in what regions are involved and in the intensity of connections that specify the network.

The individuals in each of the five functional connectivity networks also had structural, personality, and clinical characteristics that differed from each other and from healthy controls (Table 1, Supplementary Table S1). Each group represents an abnormal high-order functional connectivity network, which was later tested and confirmed to be distinguished by a distinct syndrome of self-regulatory functions and emotional reactivity measured by TCI character and temperament traits respectively along with SANS/SAPS ratings (Table 1, Supplementary Table S1).

We also compared the controls and each of the rsFC groups with one another to evaluate whether the observed rsFC differences might be confounded by differences in demographics, duration of illness, or medications used in treatment (Supplementary Tables S7, S8). We found an absence of strong effects from potential confounding variables in our young adult sample, as summarized in Supplementary Table S7 with detailed statistics in Supplementary Table S8. In each group, the average age was about 25 years, with non-significant (*p* > 0.05) and weak differences between groups (effect size *r* = .12). The gender distribution was approximately 60% male and 40% female, with 93% of the intergroup differences found to be either non-significant or weak (*r* = 0.04 to 0.31). Ethnicity was mostly white (57 to 64%) in controls and in patients in groups 1 and 4, whereas ethnicity was white in 44% of groups 2 and 5, and 30% in group 3, with some significant but weak to moderate intergroup differences (*r* = 0.11 to 0.39). Approximately 90% (range 88 to 100%) were right-handed in each group, with one weak but significant intergroup difference (*r* = 0.18). The duration of illness was similar in groups 1 - 3 (average 83 months, range 74 – 88 months) and slightly longer in the other two patient groups (103 to 104 months), but none of the differences among the five patient groups were significant (*p* > 0.05, effect size *r* = 0.12). The differences in patients use of neuroleptics, mood stabilizers, and other medications were not large, but 21% of intergroup treatment differences were significant (0.01 < *p* < 0.05) with weak effect sizes (*r* = 0.17 to 0.23).

In contrast, we found that the rsFC groups were strongly distinguished from one another by the subject’s self-reported personality features, as shown in Table 1. The power of the TCI to predict the membership of individuals in a particular rsFC group (i.e., squared multiple regression R^2^) was strong and highly significant for group 1 (R^2^ = 52%, *F* = 79.42, *p* < 1E−5, RMSE 0.11), group 3 (R^2^ = 67%, *F* = 142.9, *p* < 1E−5, RMSE = 0.07), group 4 (R^2^ = 35%, *f* = 51.2, *p* < 1E−5, RMSE 0.17), and group 5 (R^2^ = 46%, F = 7 l.9, *p* < 1E−5, RMSE 0.14). For group 2, the explanatory power was weaker due to the fuzziness of the group (R^2^ = 18.5%, F = 19.6, *p* < 3.5E−2, RMSE 0.28), but even here the TCI profiles of cases sharing features of other groups matched those of cases only in group 2 (HN, scT), as shown in Table 1. All the rsFC groups were emotionally unstable (i.e., fearful (H), impulsive (N), detached (r), and/or erratic (p)), and all had weak rational self-regulation (i.e., schizotypal or apathetic features (sc)). The significant cognitive-emotional features varied both among the rsFC groups and from healthy controls (Table 1, Supplementary Table S1). Contrary to expectations, cyclothymic personality features (sCT) did not distinguish any of the groups, and neither character nor temperament traits were differentially associated with the diagnosis (BP or SZ) of psychotic patients (Supplementary Tables S1, S3).

Based on the personality and clinical features that distinguished each group from healthy controls, we assigned descriptive names to the 5 rsFC groups to capture both motivational (subjective) and behavioral (objective) aspects of their phenotype. For example, the patients in rsFC group 1 had TCI profiles typical of avoidant personality disorder with high Harm Avoidance (H) and low self-directedness (s), indicating a disposition to defensive social withdrawal with prominent negative affect and little or no positive affect (Table 1). On the SAPS/SANS, the same subjects were described as anhedonic and asocial, so we describe individuals in group 1 as Avoidant-Anhedonic. Such close relations between the personality and other clinical variables were present for each group (Table 1; Supplementary Information: Clinical Relations distinguish rsFC groups).

### The relation of rsFC patterns to transdiagnostic clinical phenotypes

Given the strong explanatory power of personality and related phenotypes to distinguish the rsFC groups from each other (Table 1), we next tested the relationships of temperament and character to rsFC within each group. The Avoidant-Anhedonic individuals (group 1: *n* = 11, Tables 1, 2, Figs. 2A, 3A, Supplementary Table S1, Figures S1A, S2A) shared highly correlated increases in rsFC in both top-down prefrontal (DMN and FPN) and bottom-up (VAN) prefrontal networks (Table 1). This rsFC pattern was associated with defensive control, as indicated by low self-directedness (s, blaming others and feeling like a persecuted victim, *p* < 4.04E−04) coupled with significantly high Harm Avoidance (H, anxious avoidance and shyness, *p* < 3.88E−04), respectively (Table 1, Supplementary Table S1, Figures S2A-D). In addition, these three networks (DMN, FPN, VAN) also had significantly greater connectivity in these subjects than controls in two additional top-down prefrontal networks (CON and DAN) and with the visual network, but their correlations with the other components of this multi-modular functional system were less strong (Supplementary Figure 2B).

Likewise, the Sensitive-Disorganized individuals (group 2: *n* = 27, Tables 1, 2, Figs. 2B, 3B, Supplementary Table S1, Figures S1B, S2B) had increased connectivity compared to healthy controls in all six of the prefrontal networks (Table 1). This prefrontal rsFC pattern was associated with an ambivalent (HN) temperament and overattentive schizotype (scT, *p* < 1.27E-03). In addition, they had greater connectivity than controls in an extended set of functional networks including sensory networks (Visual, Auditory), Striatum, Perception, and the Somatomotor networks for control of the hand and mouth (Tables 1, 2, Supplementary Table S1, Figures S2E–H). The unique increase in rsFC of the SN and these positively coactivated networks suggests hypersensitivity to novel or unfamiliar external stimuli to which they have conflicting urges to both seek (N) and avoid (H).

The Asocial-blocked individuals (group 3: *n* = 10, Tables 1, 2, Figs. 2C, 3C, and Supplementary Table S1, Figures S1C, S2C) had a highly distinctive pattern of rsFC. In the top-down prefrontal networks, there was decreased DMN connectivity coupled with increased connectivity in the other networks (CON, FPN, DAN), associated with significant hostile mistrust (cT, *p* < 6.18E-05) and blocking. In the bottom-up prefrontal networks, SN was average, and VAN had less connectivity than controls. Other coactivated networks were more strongly connected than in controls (Auditory, Thalamus, Striatum, Context, and SM hand) whereas the visual network and the somatomotor network for the mouth was less strongly connected than in controls (Table 1, Supplementary Table S1). Networks involved in recognition of faces and social emotions (Context and Perception Networks) had greater connectivity than controls (Table 1, Supplementary Table S1, Figure S2 I–L).

The Fragile-Avolitional individuals (group 4: *n* = 35, Table 1, Figs. 2D, 3D, Supplementary Table S1, Figures S1D, S2D) had decreased connectivity in two of the four top-down prefrontal networks (CON, DMN) and increased connectivity in the other two (FPN, DAN). Functional connectivity of the CON with the somatomotor (hand and mouth), striatum, auditory, visual networks were all weaker than observed in healthy control subjects (Supplementary Table 1, Figure S2M–P), which supported the hypothesis that this configuration was uniquely low in personality measures of effortful (intentional) control and persistence (i.e., spH, *p* < 2.72E−05) (Table 1, Supplementary Table S1, Notes on rsFC circuitry).

Lastly, the Explosive-Inattentive individuals in connectivity group 5 (*n* = 16, Tables 1, 2, Figs. 2E, 3E, Supplementary Table S1, Figures S1F, S2F) had another distinct configuration. Its group members had reduced rsFC in the three prefrontal networks for sustained patterns of self-regulation (CON, DMN, DAN), consistent with an inattentive schizotype (scT, *p* < 2.60E−03) whereas the FPN, whose putative functions are task-switching and divergent thinking, was positive. Other networks were less strongly connected than in controls, including the VAN, visual, context, and perception networks (Table 1, Supplementary Table S1, Figures S2 Q–U).

### Influence of emotional reactivity on rsFC of the self-regulatory networks

Among the four top-down prefrontal networks, the FPN was consistently higher in rsFC than controls (Table 1). In contrast, the DMN, CON, and DAN varied across groups in whether they were higher or lower than controls in rsFC. Likewise, the two bottom-up networks (VAN, SN), varied in the strength of rsFC across groups. This variability allowed us to test our prediction that the bottom-up prefrontal networks were most strongly associated with temperament (Table 1, Supplementary Table S1) in such a way that fear, impulsive desire, or mistrust dominated rational self-government in patients with psychosis.

We found Avoidant-Anhedonic subjects (group 1) had high activity of the VAN associated with high Harm Avoidance (anxious avoidance, *p* < 3.88E−04), which was associated with defensive executive functioning (i.e., low self-directedness, such as blaming others as a persecuted victim, *p* < 4.04E−03). Likewise, Sensitive-disorganized subjects (group 2) had high activity of both the SN and VAN associated with both high Harm Avoidance (*p* < 7.63E−03) and high Novelty Seeking (p < 5.90E-03) coupled with overattentive schizotypy (scT, 1.27E-03 < *p* < 1.13E−02).

On the other hand, there was reduced rsFC in the VAN and average Salience in the other three groups. Asocial-Blocked patients (group 3) were Novelty Seekers (N, *p* < 3.88E-03) who were mistrustful (cT, 4.29E-02 < *p* < 6.18E-03). Group 3’s rsFC was reduced in both the VAN and DMN but increased in CON, FPN, and DAN. The Fragile-Avolitional patients in group 4 were unique in being low in persistence (p < 4.97E-03) combined with ambivalent impulses (HN, 2.72E-05 < *p* < 4.02E-02) and low self-directedness (*p* < 1.54E−03). Their temperament-character profile (spH, 2.73E-05 < p < 4.97E-03) leads to emotional fragility coupled with weak intentional control (i.e., apathy and avolition), which was associated with reduced connectivity in both the DMN and CON (Table 1). Finally, inattentive schizotypes (group 5) had explosive borderline temperaments (NHr, *p* < 8.55E-03)) coupled with inattentive schizotypal self-regulatory functioning (scT, 2.60E−03 < *p* < 5.67E−03). Their borderline cognitive-emotional profile was associated with reduced activity in the DMN, CON, and DAN but increased activity in the VAN, as expected from their intense and unstable emotional reactivity (i.e., fearful, impulsive, irritable).

### The relation of rsFC within groups to diagnoses

There were only a few weak but significant differences in rsFC between SZ and BP subjects within the groups (Supplementary Information and Table S2). For example, there were weakly significant differences (0.01 < *p* < 0.05) by diagnosis in network connectivity correlations in group 1 (4 of 279), group 2 (12 of 1077), group 3 (3 of 366), group 4 (0 of 627) and group 5 (1 of 209). Further details are presented in Supplementary Information (Relations of features within groups to diagnosis, Tables S1-6).

### Structural connectivity distinguishes each functional connectivity group

**Fig. 4:**
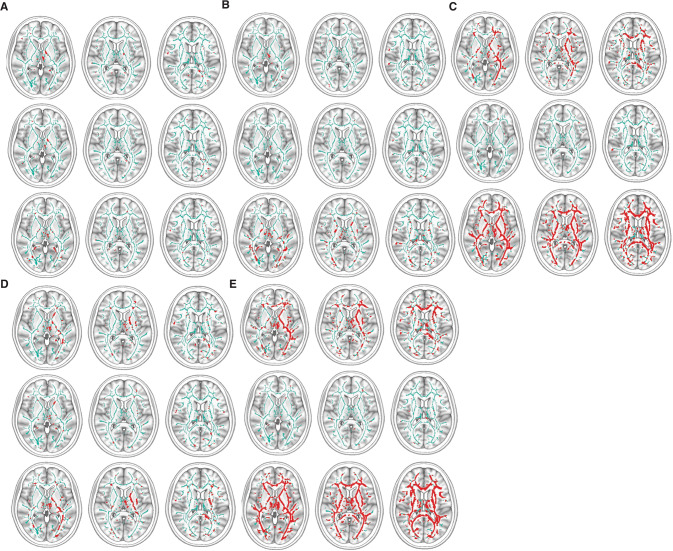
TBSS low FA images corresponding to each rsFC group. Low FA is red when *p* < 0.05 adjusted for multiple comparisons of voxels. The images are arranged for the whole group (top row), BP subjects (middle row), and SZ subjects (bottom row) of each group. **A**–**E** represent each of the five sets that describe different patterns of connections among the nodes: **A** the Avoidant-Anhedonic (group 1), **B** the Sensitive-Disorganized subjects (group 2), **C** the Asocial-Blocked subjects (group 3), **D** the Fragile-Avolitional subjects (group 4), and (E) the Explosive-Inattentive individuals (group 5).

Decreased fractional anisotropy (FA) maps of white matter microstructure distinguished the five groups of subjects that we identified based on their rsFC alone (Fig. 4, Supplementary Table S1). The FA pattern for each group was distinct, but all of them had significantly asymmetric involvement, more severe in the left hemisphere. White matter tracts associated with the different rsFC groups primarily involved prefrontal fibers and their connections to motor, limbic, and cerebellar regions.

For most components of each rsFC group, we observed corresponding white matter structural abnormalities, as shown in Fig. 4 and detailed descriptions in Supplementary Information and Table S1. For example, members of each group had uniquely low FA in specific white matter tracts underlying its rsFC, including group 1 (fornix), group 2 (superior corona radiata, inferior and middle cerebellar peduncles), group 3 (superior cerebellar peduncle), group 4 (thalamic radiation to the posterior limb of the internal capsule), and group 5 (cingulum, superior longitudinal fasciculus) consistent with their rsFC (Supplementary Information on structural connectivity of each rsFC Group, Table S1).

## Discussion

### Four major findings

Our most important findings are fourfold. First, we confirmed a well-established observation that is essential to keep in mind: *human brain functioning involves the collaboration of multiple distributed and complex adaptive networks, not discrete or localized centers*. In healthy individuals, the collaboration of these systems is harmoniously orchestrated, whereas in patients with psychoses and related personality disorders, they are discordant— habits, goals, and values are discordant because negative emotions and irrational thoughts overwhelm weak and dysfunctional self-regulation. Extensive research has shown that the neural assemblies that comprise these distributed functional connectivity networks are synchronized to work collaboratively by a combination of neurobiological and psychosocial mechanisms [135–139].

Second, *stable outpatients with psychotic disorders (BP or SZ) differ strongly from healthy controls in the organization of both their personality and their brain functional connectivity*. Among stable outpatients with psychosis (BP or SZ), we identified five groups of patients with distinctive patterns of rsFC measured by protocols standardized for the Human Connectome Project. The identified groups were then validated by independent measures of structural connectivity, personality, and other clinical features not used to identify the rsFC groups. These groups were distinguished by configurations of rsFC networks related to specific biopsychosocial aspects of psychosis: sensory hypersensitivity, negative emotional balance, impaired attentional control, avolition, and social mistrust. Vulnerability to psychosis was associated with these dysfunctional configurations of rsFC networks, particularly impaired connectivity of the prefrontal cortex with parietal, temporal, limbic, and cerebellar hubs.

Third, we found that *variable configurations of rsFC are internal representations of a person’s temperament and character organization*. The four top-down prefrontal networks (DMN, CON, FPN, DAN) mediate mental self-government as measured by TCI character traits, whereas the two bottom-up prefrontal networks (VAN, SN) mediate sensory and emotional reactivity as measured by TCI temperament traits. Both rsFC and personality configurations represent the same underlying complex systems of learning and memory that are meta-stable and trait-like, but not fixed or discrete [8, 12, 66–68]. We found that temperament and character explained most of the variability in rsFC configurations that was possible (i.e., squared regressions of 35% to 67%) when considering the test-retest correlations indicating the reliability of the measures of personality (~0.6 to 0.8) [15, 76] and rsFC (-0.8 to 0.9) [66].

Fourth, *stable outpatients with schizophrenia were highly similar in their rsFC and personality to those with bipolar disorder despite the patients with different diagnoses having distinct signs and symptoms when acutely psychotic*. We had identified vulnerability to psychosis by identifying the configurations of rsFC among stable outpatients with diagnoses of either schizophrenia or bipolar disorder compared to healthy controls (see third finding). However, we found nearly equal numbers of patients with each diagnosis in every rsFC group. Also, there were only a few weak but significant differences by diagnosis in the network connectivity correlations in every rsFC group.

These finding combine to support a diathesis-stress model that postulates largely different mechanisms for diathesis (i.e., predisposition) to psychosis and for stress reactivity that provokes the diagnostic signs and symptoms of different forms of acute psychosis. In the next section we discuss how all four of our major findings contributed to uncovering a more refined diathesis-stress model of psychosis than has been possible previously.

### A diathesis-stress model of functional dysregulation in psychosis

Health can be characterized by integration of biopsychosocial functions so that a person’s habits, goals, and values are in accord with one another in promoting their well-being [85, 140–142]. In major findings two and three of this study, we showed that temperament-character profiles and/or rsFC can be used to distinguish between healthy people and those predisposed to psychosis. The widespread synchronization of the oscillatory activity of rsFC networks allows widely distributed communication throughout the brain, promoting efficient collaboration in diverse biopsychosocial brain functions, as noted in our first major finding. This synchronization is facilitated by coordination of multiple neurobiological and psychosocial mechanisms [135–139]. The observed neural mechanisms of synchronization of neural assemblies include synaptic transmission of inhibitory and excitatory impulses that lead to coupling of neural assemblies locally and distributed at a distance, the synchronization of groups of neurons with similar preferential firing rates, and gap junctions for inhibitory synaptic transmission that initiate firing of many neurons at once upon cessation of inhibition. In addition, synchronized neural networks and related personality configurations are facilitated by mutual constraints on activity by focusing activity on shared goals, shared interpersonal experience in dyads and larger social and ecological settings [135, 143]. These psychosocial influences serve to synchronize neural networks while promoting mutual trust and respect under favorable conditions, such as rearing with parental warmth and tolerance, which is positively selected in evolution by promoting longevity and reproductive fitnesss [12, 13]. In combination, neurobiological and psychosocial mechanisms can reinforce one another to promote biopsychosocial integration at intercellular, individual, social, and ecological levels of synchronization.

Genomic studies have identified both genetic and environmental variables that influence the development of personality, including temperament, character, and their joint integration [12, 13, 56–58]. Different molecular pathways influence complex systems of learning for behavioral conditioning, intentionality, and self-awareness, which correspond to distinct configurations of temperament and character [58]. Our second major finding indicates that stable outpatients with either schizophrenic or bipolar psychoses have impaired rsFC that is associated with strong irrational emotional drives that bias and distort their perceptions, expectations, intentions, and values so much that self-government become unrealistic and maladaptive. Likewise, our third finding indicates that individuals with extreme temperaments and weak character development are vulnerable to psychosis. For example, selfish desire and hostile mistrust (N and cT, as in groups 2, 3 and 5) impairs the legislative functions of cooperation and leads to schizotypal outlooks of separation with magical thinking (scT). Fearful avoidance (H and s, as in groups 1 and 4) impairs the executive functions of self-direction and intentional control. The path to psychosis involves the false perception of separation from other people and things: apathy and hopelessness (as in avoidant and apathetic personalities) or mistrust and grandiosity (as in schizotypal personalities).

However, as shown by our third major finding in stable outpatients, people with a vulnerability to psychosis do not always manifest their predisposition continuously or ever, as is clearly demonstrated in neurocognitive studies of monozygotic twins discordant for psychosis [144]. Likewise, not all people with schizotypal characters (scT) and ambivalent (HN) or explosive (rHN) temperaments develop psychosis [145]. The integration of human brain functions for predisposition to well-being occurs in the neocortex and its cortico-thalamo-cortical loops [136], particularly the prefrontal cortex and its reciprocal functional and structural connections with other regionally distributed hubs [39].

Our fourth major finding supports the hypothesis that the level of stress reactivity that precipitates or exacerbates acute psychosis involves dysfunctional regulation of internal and external stressors. The neural diathesis-stress model postulates that the hypothalamic-pituitary-adrenal (HPA) axis regulates stress-reactivity related to psychosis and that cumulative stressors impair functional connectivity, and eventually lead to dopaminergic sensitization that impairs higher cognitive and temperament-character functioning through cortico-striatal activity [146–149].

The brain regulatory system that regulates stress reactivity is a component of a more general system of automatic and instinctive predictive regulation to maintain homeostasis and allostasis in animals. Allostasis is the predictive regulation of adaptive responses that maintain health by anticipating changing or stressful conditions [150–153]. This stress-responsive system is integrated by regulatory functions of the hypothalmus [98], with its light-responsive suprachiasmatic nucleus to synchronize systems in every tissue through the HPA axis, thereby anticipating and coordinating behaviors to secure future needs, while rewarding better-than-predicted results with dopaminergic stimulation [137, 152, 153]. The hypothalamus controls basic life functions of all internal organs through the autonomic nervous system, as well as cells groups in the brainstem that control autonomic reflexes, including breathing, which are instinctive [154]. Instinct refers to adaptive behaviors that are genetically determined, innate, reflexive, unlearned, and stereotypic in response to anticipated needs and external stressors [155]. In addition, instincts are automatic reflexes that do not depend on conscious thought (i.e., habits, intentions, reasoning, or self-awareness). Instinctive behaviors related to sex, hunger, thirst, wakefulness, defenses against attack (fight, flight, freeze) and other activities related to maintenance of health, survival, and reproduction are observed in all animals, including humans [156].

The hypothalamus is the highest level of integration of regulatory brain functions in reptiles [98]. In mammals, it is part of the multi-level limbic system in which both instinctive and learned emotional behaviors can be integrated with a person’s goals and values through reciprocal connections with the prefrontal cortex of healthy people [98]. However, human instinctive behaviors become dysregulated during acute psychosis, leading to extreme displays of sexuality, consumption, activity, wakefulness, and basic emotions (fear, anger, disgust, elation, sadness) in BP, defense against attack, perseverative and stereotypic behaviors in SZ, or mixtures of these in some psychoses and other disorders [157, 158]. Displays of such instinctive behaviors tend to increase in frequency along with development of positive psychotic symptoms [145] when recurring stressful experiences create a vicious cycle of increasingly dysfunctional regulation and more stress [147].

More generally, instinctive responses to physiological challenges and stress throughout the body are genetically regulated by non-coding RNAs, particularly lncRNAs and microRNA, in all cellular life forms with or without complex nervous systems [159–162]. The regulatory ncRNAs have many essential regulatory functions including transcriptional and post-transcriptional regulation of gene expression and co-expression, splicing, translation and post-translational modification, assembly of large multiprotein complexes, and epigenetic modification of DNA. Regulatory RNAs operate within cells and are also released in extracellular vesicles as chemical messengers for cell-cell communication, just as hormones of the HPA axis are also secreted in vertebrates [163]. The cellular and extracellular functions of regulatory RNAs permit organisms to operate as dynamically self-organized multi-component ensembles that adapt automatically to changing conditions and stressors without reliance on conscious thought.

In humans this reflexive-instinctive level of adaptation can collaborate with the higher level of conscious adaptation because ncRNAs are also involved in regulation of temperament and character [12, 13, 56–58]. However, in people predisposed to psychosis by personality disorder, or those exposed to repeated trauma, neglect, and stress, particularly in childhood, or following extensive use of intoxicants, this communication and collaboration is impaired, thereby leading to increased vulnerability and dysregulated stress reactivity [147]. Common adverse stressors that precipitate or exacerbate psychosis include traumatic and adverse life events associated with urban environments, migration, military activity, disasters, or loss of family and friends, which may be proxy measures of fear, violence, and social isolation and inequity leading to perceptions of being separate or an outsider without control, support, or trust [147, 164]. Therefore, among stable outpatients with psychosis, as observed in this study, the association between specific diagnoses based on signs and symptoms of acute psychosis (BP or SZ) is expected to be only weakly correlated with their predisposition to psychosis, as measured by brain functional connectivity or self-reported personality configurations.

In addition to our current findings, the proposed biopsychosocial diathesis-stress model explains several other important findings from prior independent research. The hypothesis that there are distinct complex genetic systems that regulate predisposition and stress reactivity also provides an explanation for the variable natural history of psychoses, such as progression with repeated adverse events [147] or improvement under favorable conditions [85]. In addition, there is moderate specificity in the types of childhood adversities reported by patients with schizophrenia spectrum disorders and mood disorders: the stressors in the schizophrenia spectrum are predominantly the presence of unstable and uncaring relationships with much abuse and neglect, whereas the stressors for bipolar disorder and major depressive disorders are predominantly absence or loss of stable caring relationships [165].

Further tests of the proposed diathesis-stress model in transcriptomic studies are underway. We have already carried out studies of personality in relation to the human genome, but not with its transcriptome. The human genome is composed entirely of DNA with nucleotide sequences for a little more than 63,000 genes in total. Just under 20,000 of these sequences code for proteins and most of the other 43,000 are transcribed to ncRNAs [166], which regulate adaptation to changing intracellular and extracellular conditions. The most prominent biomarkers of acute disease states are these transcribed regulatory ncRNAs [162]. Accordingly, we have begun transcriptomic studies that may allow us to characterize the organization of these genetic transcripts to clarify the regulatory control of stressful and destabilizing events.

### Strengths and limitations

A possible limitation of our work is that our sample size was not large enough to allow dividing it into an initial calibration sample and a validation sample. Nevertheless, our sample size was highly informative to identify the large differences among patients in the different functional connectivity groups. Exploratory analyses do require validation, which we did by testing the differences between the functional groups using information independent of that used to select subjects or to identify the functional connectivity groups.

A strength of this approach to validation in moderate sized samples is that it illustrates the value of thorough phenotyping, which allowed us to have a deep understanding of the neuroadaptive processes that account for their clinical characteristics. We suggest that the thorough assessment of moderate-sized samples is particularly important because it allows detection of strong effects with broad coverage, which in turn facilitates future research and translation into useful clinical practices.

### Implications of a biopsychosocial approach to human brain functions

Our findings indicate that human functioning cannot be reduced to physical processes, to psychological processes, or social processes – all three aspects of human functioning collaborate interdependently, as illustrated here in brain functional connectivity. *A biopsychosocial diathesis-stress model provides a conceptual framework that can guide an understanding that respects the utility of traditional clinical diagnoses along with the causal and developmental insights that can be gained from both neurobiological and psychosocial measures*.

There has been a growing crisis of confidence about the validity and utility of traditional diagnostic categories in psychiatry represented in DSM and ICD, including many calls for a paradigm shift due to pervasive comorbidity and failure to identify specific objective laboratory tests to confirm specific diagnoses [167]. Likewise alternative proposals for bottom-up approaches like RDoC that seek to begin with basic neurobiological findings to explain clinical phenomena are insufficient when applied to the complex adaptive systems that underlie common health problems [167, 168]. We suggest that the controversy about the validity and utility of traditional clinical diagnoses may be due to a misunderstanding that is much like earlier controversies about development and regulation of the effects of nature versus nurture in biology, diathesis versus stress in psychology, and person versus situation in personal and social fields of science. Common disorders in medicine, regardless of specialty, involve a hierarchy of complex adaptive systems with fuzzy boundaries, so dysregulation and dysfunction are not expected to fall neatly into separate diagnostic categories or separate centers in the brain or other organs.

Nevertheless, the strong relationship between brain functional connectivity and personality in humans can help to bridge the explanatory gap about the meaning of neuroimaging data and self-reports of the biopsychosocial aspects of human personality. As recommended in recent reviews of alternative perspectives on psychiatric validation [169], this finding advances the program of validation in psychiatry in which objective neurobiological findings can be translated into meaningful and clinically relevant descriptions of concurrent human behavior and subjective experience, and vice versa. Fortunately, we are positioned to begin to address such challenges by integrating prior findings about the genetics, development, and evolution of human personality with our current findings on the hierarchical organization of brain functional connectivity.

### Supplementary information

Supplementary Information

Supplementary Figures S1-S11

Supplementary Table S1

Supplementary Table S2

Supplementary Table S3

Supplementary Table S4

Supplementary Table S5

Supplementary Table S6

Supplementary Table S7

Supplementary Table S8
