# Supplementary material for: Temperament & Character account for brain functional connectivity at rest: A diathesis-stress model of functional dysregulation in psychosis
Source: Mol Psychiatry. 2023 Apr 4;28(6):2238–53. doi: 10.1038/s41380-023-02039-6 (PMC10611583; doi:10.1038/s41380-023-02039-6)
Supplement: Supplementary file 1 — Supplementary Information [file 41380_2023_2039_MOESM1_ESM.docx]

**Supplementary Information**

**Clinical validation of fMRI groups**

**Methods and Materials**

**Rationale of the methods**

Good clusters instead of good clustering

Clusters vs biclusters

Clusters and graphs

Multi-view biclustering selection

**Specific description of the methodology**

(1) Preprocessing datasets by flattening matrices

(2) Identifying optimal functional connectivity sets by multilevel NMF factorization:

*(2.1) Mathematical description of the NMF*

*(2.2) Decomposing the data into a multilevel family of sub-matrices*

(3) Dissecting factors in biclusters

*(3.1) Dissecting NMF k factors into sub-matrices or biclusters which are interpreted as fMRI sets*

*(3.2) Learning the W and H matrices of FNMF*

(4) Selecting biclusters: multi-view and optimally assembling the families of sub-matrices

(5) Statistical analysis of biclusters

(6) Graph and matrix representations of biclusters

*(6.1) Displaying biclusters extracted after factorization*

*(6.2) Displaying and decoding TBSS biclusters and transforming them back to native space*

*(6.3) Displaying and decoding fMRI biclusters and transforming them back to native space*

**Notes on Connectivity Circuitry**

**Clinical features distinguish each rsFC group**

**Relations of rsFC and other features within groups to Diagnoses**

**Structural connectivity of each rsFC group**

**Table and Figure Legends**

**Clinical validation of fMRI groups**

In addition to SAPS/SANS ratings that described significant differences between the composite groups of BP and SZ patients used in naming the groups, there were some additional differences between BP and SZ patients both between and within groups (Tables 2, S1, S2, S3). Specifically, Avoidant-Anhedonic patients in group 1 as a whole were distinguished by anhedonia and social withdrawal from the other groups, and this difference was significant in the SZ patients alone but not in the BP patients alone. Sensitive-Disorganized patients (group 2) were distinguished as a whole by their disorganized SAPS features (bizarre behavior and formal thought disorder), whereas the SZ patients in group 2 were further distinguished by flat affect, alogia, anhedonia, and attention. Asocial-Blocked subjects in group 3 as a whole were distinguished by Thought Blocking and Somatic Delusions, whereas only SZ patients in that group had abnormal attention and only BP subjects had inappropriate affect and distractibility (Tables S1, S2). Both BP and SZ patients in group 4 were distinguished by avolition, and SZ patients in that group were also distinguished by hallucinations, delusions, and attention. In group 5 as a whole, Disorganized-Inattentive subjects were more inattentive as a whole than those in other groups, but the difference was significant in SZ patients alone and not in BP patients alone.

Regardless of diagnosis (BP or SZ), Sensitive-Disorganized patients (group 2) had more significant differences from controls on the Young Mania Rating Scale (YMRS) than did than all patients combined (Tables 2, S5). We found that Sensitive-Disorganized subjects (group 3) showed more significant differences from controls than the full set of BP+SZ patients, and this was consistent in both BP and SZ patients within this group. The Avoidant-Anhedonic subjects (group 1) showed similar differences, but mainly due to the BP patients.

Estimates of chronic psychotic and affective symptoms were derived using the Washington Center for Early Recognition Psychosis and Affect Screen (WERCAP, Tables 2, S5). Some groups had more highly significant differences in WERCP psychosis scores were more highly significant from controls than were the full set of patients. These included SZ patients from the Avoidant-Anhedonic group 1 and the Fragile-Avolitional group 4, and BP patients from Asocial-Blocked group 3, Fragile-Avolitional group 4, and Disorganized-Inattentive group 5. SZ patients in the Fragile-Avolitional group 4 showed these differential values ​​based on WERCAP mania scores. As in the case of the global SAPS or SANS, most groups show significant differences from controls in all YMRS and WERCAP clinical characteristics, and the more significant differences (p-values) in some groups indicate what best distinguished that group from the others.

**Methods and Materials**

Diffusion tensor imaging (DTI) scans and analysis based on Tract Based Spatial Statistics (TBSS) and Non-negative Matrix Factorization (NMF) of neuroimages was performed as described in ^1^. The NMF-based DTI-TBSS Analysis (NDTA web server http://picu.ugr.es/ndta/) method for structural connectivity was customized for 2D matrices of functional connectivity as described in the following sections (Figs. S6-S7). Further methodological descriptions of PGMRA used for different domains of knowledge are available in ^2-11^, and its web server application is online at <http://phop.ugr.es/fenogeno> ^7^. Fast parallel software implementations were run at the Center for High Performance Computing (CHPC) and RIS facilities at WUSM.

**Rationale of the methods**

**Good clusters instead of good clustering**

We are focusing on identifying good clusters instead of good clustering (Fig. S5) ^9-14^. Good clusters can be identified without choosing *a priori* a particular number of maximum clusters ^15-19^. Setting this parameter as a small ^19^ number may eventually generate few but large data partitions here defined as general clusters that may underfit the data. In contrast, using a big number of clusters will generate many clusters with partitions of small size (i.e., more granular partitions) here defined as specific clusters that may overfit the data but will probably reveal true properties of the uncovered object (e.g., biological knowledge) ^3, 16, 18, 20, 21^. Although there are many different validity indices that suggest the best number of clusters for a given dataset, they often produce contradictory results ^14, 22^. This problem is exacerbated when Centroid‑based (e.g., k‑means, NMF) or Distribution‑based clustering (e.g., EM) approaches are utilized because two successive numbers of clusters may generate completely different cluster arrangements (see ^14, 22^ for a review).

Establishing the optimal number of clusters is an unsolved computational problem since different features emerge from different assumptions about this number ^15-18^(Fig. S5). Instead of dealing with different and controversial results often obtained from optimizing validity indices ^14, 22^ that indicate a single number of clusters, we calculated clusters from partitions generated by all number of clusters between 2 and √𝑛 ^22^, where *n* is the number of observations (subjects). We postponed the selection of the best clusters until all multilayer partitions were examined to select a set of clusters that together provide an optimal description of the sample. These clusters could be chosen from different partitions that were generated by different number of clusters (Fig. S5, see Consensus clustering ^15-18, 21, 23^).

**Clusters vs biclusters**

Our method pursuing local partitions of brain images provides substantial advantages over classical clustering approaches (Figs. S6-S7). Averaging and comparing groups would be expected to miss real differences in connectivity if they are localized in different places in each patient (Fig. S7F). In contrast to classical clustering techniques, such as hierarchical clustering from Sokal and Michener (1958) and k-means clustering, from Hartigan and Wong (1979), we used biclustering techniques that do not require patients in the same cluster to perform similarly over all connections (Fig. S5). Biclusters are defined in graph theory as bipartite graphs with maximal cliques (see below). Classical clustering methods derive a global model whereas biclustering algorithms produce a local model in which signals emerge only in relevant dimensions. In sum, we look for good clusters instead of good clustering ^9-14^.

**Clusters and graphs**

In the mathematical field of graph theory and computer science, an adjacency matrix is a square matrix used to represent a finite graph (Fig. S8A). The elements of the matrix indicate whether pairs of vertices are adjacent or not in the graph. Every matrix has its corresponding graph and vice versa (Fig. S8A). A bipartite graph (or bigraph) is a graph whose vertices can be divided into two disjoint and independent sets *U* and *V*, that is every edge connects a vertex in *U* to one in V (Fig. S8B). A clique, is defined as a subset of vertices of an undirected graph such that every two distinct vertices in the clique are adjacent (Fig. S8C). Biclusters are bipartite graph with maximal cliques, where *V* and *U* may represent subjects and features, respectively, which are clustered by their connection through edges ^24^ (Fig. S8D). For example, Fig. S8D shows a bipartite graph G1 that has an edge maximum biclique B1({u1,u2},{v1,v2,v3}) with 5 vertices and 6 edges, and a vertex maximum biclique B2({u3,u4,u5,u6,u7},{v5}) with 6 vertices and 5 edges. Both B1 and B2 are maximal. Methods that pre-imposed a number of clusters, as well as a full and crisp membership of the observations to them, add edges where they do not exist.

**Multi-view biclustering selection**

Our method uncovers multi-view and optimal biclusters defined in distinct granular partitions (e.g., multi-faceted/hierarchical tensor decomposition ^25^) from distinct number of clusters without being exhaustive or redundant. Optimality was defined as a trade-off among specificity, generality, and diversity of the biclusters by multiobjective optimization ^3, 13, 26, 27^. Here the assumption is that biclusters generated with a small maximum number of clusters k (low granularity) tend to include a large number of subjects, and thus, they are likely to share a more heterogeneous set of features (e.g., functional connections or voxels with lowFA values) than a smaller group of subjects from a cluster generated when using a large k-value (high granularity).

**Specific description of the methodology**

**(1) Preprocessing datasets by flattening matrices**

*Organizing data as a 2^nd^ order tensor (Fig. S6):* For fMRI connectivity, each subject was represented by a 369x369 2D matrix built with pairwise fMRI connectivity correlation values between brain Gordon networks ^28^. Negative correlations were not considered. Each connection-correlation value in a 2D matrix corresponding to a subject was preprocessed to facilitate the entire factorization of matrices corresponding to all subjects with the Non-Negative Matrix Factorization (NMF) method by using:

*((CorrelationValue – CorrelationControlAvg) +1)^2^* ***(eqn. 1)***

where the average of all controls was considered the “normal” correlation value for a given connection. Each connection value was then unfolded into two variables: above (high) and under (low) the control average to keep track of each type of differential value. Then, 3^rd^ order tensors, combining information across subjects (i.e., values x connections x subject), were flattened into a 2^nd^ order tensor that constituted the input of the NMF factorization method. The output of the NMF method included a decoder that showed factorized results as heatmaps based on the original connection-correlation values. Factors with less than 4 subjects, or only one connection between network parcels were excluded.

Graph representation of connections includes nodes corresponding to a network (i.e., Auditory, Visual…), and edges (i.e., linkages), corresponding to the number of connections between the nodes (network parcels, Fig. S9A). Blue and red edges represent correlation values above and below control average, respectively. Note that a single network (e.g., Auditory) represents multiple sub-network connections (e.g., Auditory_1, Auditory_2, etc., Figs. S9B-D). Each column and row in the matrix corresponds to a network (i.e., Auditory, Visual…) where a cell encodes the number of connections between the nodes of networks (network parcels). Again, cells are color-coded as graphs (Fig. 9C).

DTI images were encoded as described in ^1^. For each subject, white matter FA voxels extracted from TBSS images were organized into a 3^rd^ order tensor ^25^ (i.e., x, y and z dimensions), using *oro.nifti* package, R version 2.15.1. The 3^rd^ order tensors were structured into 4^th^ order tensors by combining information across subjects (i.e. x, y, z, and subject dimensions). The 4^th^ order tensor was then flattened into a 2^nd^ order tensor (i.e. voxel and subject dimensions) by collapsing its three spatial dimensions into a single dimension. 126,586 voxels were present in the TBSS skeleton, and the rows corresponding to the voxels outside the TBSS skeleton were removed from the matrix. Finally, the matrix was normalized by scaling each row in the [0,1] interval (i.e., 1-((x-xmin)/(xmax-xmin))).

**(2) Identifying optimal functional connectivity sets:**  **multilevel NMF factorization**

*(2.1) Mathematical description of the NMF (Fig. S7A-B):* We consider a dataset consisting of a collection of n subject samples, which we use to characterize a domain of functional (fMRI) or structural (DTI) states of interest. Here, we illustrate the NMF by the fMRI type of data, but it can be extended to any other type of data. DTI data was extensively described in ^1^ and implemented as a web server (http://picu.ugr.es/ndta). The data are represented as an *m* x *n* matrix X, whose rows contain the correlation values of the *m* brain networks in the *n* subject samples. Using NMF, we find a manageable number of factors *k*, positive local and linear combinations of the *n* subjects and the *m* connections, which can be used to distinguish the functional connectivity profiles of the subtypes contained in the dataset. Mathematically, this corresponds to finding an approximate factoring, *X_mxn_* ~ *W_mxk_* x *H_kxn_*, where both matrices have only positive entries and hence are biometrically meaningful ^7, 29-31^. *W* is an *m* x *k* matrix that defines a decomposition model whose columns specify how much each of the subjects contributes to each of the *k* factors. *H* is a *k* x *n* matrix whose entries represent the correlation values between brain networks of the *k* factors for each of the *n* subject samples. In our implementation either a subject or a connection can belong to more than one factor ^4, 7, 21^.

*(2.2) Decomposing the data into a multilevel family of sub-matrices (Fig. S7B).* Our generalized factorization method applies a basic factorization method recurrently to generate multiple matrix partitions using various initializations with different maximum numbers of sub-matrices *k* (e.g., $2\leq k\leq\sqrt{n}$ where *n* is the number of subjects), and thus, avoids any assumption about the ideal number of sub-matrices (see ^7^ and ^1^ Supplementary material) for a rationale about the use of unconstrained number of sub-matrices or clusters). Specifically, we use FNMF as described in the prior section. For each run of the basic factorization method ($2\leq k\leq\sqrt{n}$), all sub-matrices are selected to compose a family of fMRI sets sets *G_k* ={G_*k_i*}, where $1\leq i\leq k$. Each *G_k* family, as well as all families together *G* ={G_*k*} for all *k*, may include submatrices (i.e., sets) that are overlapping, partially redundant, and different in size.

**(3) Dissecting factors in biclusters**

*(3.1) Dissecting NMF k factors into sub-matrices or biclusters which are interpreted as fMRI sets (Fig. S7A)*: Each *k* factor has the same size of the original *X* matrix, and this matrix is reconstructed by the sum of the subjacent matrices of these *k* factors (see above, and Fig. S7). To identify the useful information that characterizes each factor and distinguishes it from the others, we decompose the factorial matrix using a bipartite graph with maximum representation of cliques (which are more generally called biclusters). The original bioNMF method ^30, 32^  uses the non-smooth variant of the NMF algorithm (nsNMF). This variant achieves an easier interpretation of the factors (*k*) due to the intuitive sparse, non-overlapped part-based representation of the data. Once the *W* and *H* matrices are calculated, the method selects the most representative features and observations (subjects) for each factor in order to build the biclusters. The bioNMF algorithm defines the factor-specific rows or columns as those rows or columns in the *H* and *W* matrices, respectively, that show high coefficients for a given factor, as well as low coefficients for the other factors. Given a certain factor *k*, i.e., the lt^h^ column of *W*, all features in the dataset can be properly sorted by their association to the local pattern captured by this factor (Figure S7A(ii-iii)). At the same time, observations/subjects can also be sorted by their coefficients in the corresponding factor, that is, the l^th^ row of H. This operation is carried out in one-to-one correspondence among columns of *W* and rows of *H*, generating *k* natural ordinations of the matrix in which features and subjects highly related in a sub-portion of the data. The set of selected rows and columns for each factor define a bicluster.

We developed a fuzzy variation of the bioNMF biclustering method named Fuzzy NMF or FNMF ^7^, where every column or row can belong to many biclusters or, eventually, to all of them ^4, 6, 21^. In addition, our FNMF includes a strategy to identify and discard outliers from the biclusters, as in a possibilistic clustering method ^6, 14, 22^. Unlike the bioNMF biclustering method, our FNMF analyses each factor by selecting the rows or columns with the highest values based on a threshold established as an input parameter. This threshold indicates the level of fuzziness, and in turn, which values will belong to a bicluster. The threshold is defined in the unit interval [0-1]. For example, the threshold for factor *i* in the matrix *H* is calculated as:

*Threshold = max ( H_i_ ) * (1 – fuzziness)* ***(eqn. 2)***

where all values above the threshold will be kept in the bicluster. The selection process for one factor takes into account only the values within that factor (that is, they are independent of the values of the rest of the matrix, Figure S1D(iii)). The fuzziness allowed in the current study was 30%. From now on, we will use the terms sub-matrix, bicluster, or set (SNP or phenotypic) as synonyms that serve to emphasize specific features of the same thing in different analytical or clinical contexts.

*(3.2) Learning the W and H matrices of FNMF:* Due to the non-deterministic nature of FNMF, it may not converge to the same solution on each run because of the random initial conditions used. Therefore, we execute the algorithm 40 times, as was originally suggested for the bioNMF algorithm ^7^, with different random initializations for selecting the *W* and *H* matrices that best approximate the input matrix. FNMF makes use of the convergence method described in ^33, 34^ to establish the stopping threshold that controls the algorithm convergence on each run. Each 10 iterations, a connectivity matrix *M* of size *C* × *C* is computed, where *C* is the number of columns of matrix *H*. Each entry *M_ij_* in this matrix is set to 1 if column *i* and *j* in *H* have their maximum value for the same factor (i.e. on the same row in *H*), and 0 otherwise. If the connectivity matrix stops changing after a certain number of iterations (which equals the stopping threshold multiplied by 10), the matrices are considered as having converged and the algorithm stops the current run. The learning process of the *W* and *H* matrices is performed with projected gradient descent methods ^33, 35^.

**(4) Selecting biclusters: multi-view and optimally assembling the families of sub-matrices (Fig. S10).** Because sub-matrices can be defined at different levels of granularity, we apply a competitive learning approach (i.e., consensus clustering ^19 , 20^) to select and assembly optimal, non-redundant, and cohesive sub-matrices using multiobjective and multimodal optimization techniques ^3, 13, 26, 27^. Optimal sub-matrices were obtained as a tradeoff between two opposing objectives: sensitivity and generality ^4, 6, 8, 13, 21, 26, 27^. Sensitive sub-matrices tend to be composed of few observations (i.e., subjects) described by multiple features, whereas specific sub-matrices are composed of many observations described by few features ^12-14, 21, 22^. A Pareto-optimization strategy searches for solutions that are non-dominated in the sense that there are no other solutions superior in all objectives being selected (i.e., close to the Minimum Description-Length (MDL) ^36^). The dominance relationship as a minimization problem is defined as:

$a\prec b \text{iff} \forall i O_{i}\left( a \right)\leq O_{i}\left( b \right) \exists j O_{j}\left( a \right)<O_{j}\left( b \right)$***(eqn. 3)***

where the *O_i_* and *O_j_* are either specificity or generality objectives. Optimization of small sets of sub-matrices was exhaustively implemented, whereas evaluation of large sets is approached by Genetic Algorithms, as described in ^5, 6, 37^ (Fig. S10C). Multiobjective and multimodal approaches constitute the basis of multi-view ^38^ machine learning provides a multifaceted recognition of new objects described from different perspectives. Another indirect objective considered for the evaluation of sub-matrices is the generation of diverse patterns that completely describe objects (subjects). Therefore, our approach evaluates the sensitivity and generality objectives described above in a local niche ^13, 21, 26, 27^ (Fig. S10B-E).

In the fMRI domain, both sensitivity and specificity measurements are based on counting either #subjects or #connections within a sub-matrix or bicluster without distinguishing among them (Figs. S10-S11). However, diversity qualitatively differentiates which subjects and/or connections make up a bicluster, and therefore different qualitative biclusters are assigned to different niches. These niches are calculated using Jaccard’s metric between sub-matrices ^5, 37^ (i.e., inclusion of subjects and connections, Fig. S10):

${Niching(B}_{i},B_{j})= \frac{{S(B}_{i}) \cap{S(B}_{j})}{{S(B}_{i}) \cup{S(B}_{j})} >\gamma$***(eqn. 4)***

where *B_i_* and *B_j_* were two different sub-matrices, the *S* functional retrieves the subjects and connections in the sub-matrices in a particular niche, and $\gamma$ is the size of the niche determined by the degree of overlap (here, $\gamma$ = .5 including both subjects and connections) between sub-matrices (Fig. S10). Here the assumption is that the niches are equivalence classes dictated by the degree of overlap/inclusion between subjects in the sub-matrices.

In sum, biclusters compete with each other if and only if they are in the same niche (Fig S10A). For example, given two sub-matrices where one of them has the same or even worse sensitivity and generality than the other but correspond to different sets of subjects, both sub-matrices will be preserved because they are in different niches. The pseudocode is shown in ***(eqn. 5)*** and niches for the 5 selected biclusters are illustrated in Fig. 11:

$Given a set of biclusters B=\left\{ b_{1,}\ldots,b_{n} \right\}; \forall i,j;$ ${\text{if} Niching\left( b_{i,}b_{j} \right) \& \left( j<n \right) \& (\text{if} b}_{j}\prec b_{i}) deleteb_{j} from B;opponent= b_{j+1};Else\text{if}\text{ (j<n)}$ $\text{opponent=}b_{j+1};Else\text{if}\text{ }\text{}\text{ (}b_{i}\prec b_{j}\text{ OR }b_{j}\prec b_{ij}\text{) } opponent= b_{j+1}\text{; Else Select }b_{i}; i=i+1$***(eqn. 5)***

**(5) Statistical analysis of biclusters**

Statistical significance was assessed by comparing the connections characterizing each bicluster as measured by the correlation coefficient with the same connectivity values of subjects not involved in that bicluster, including comparisons with controls and/or subjects in other biclusters, using one-way ANOVA and pairwise t-tests (R version 2.15.1) and applying Bonferroni correction (Fig. S2). Significance in each domain of knowledge, including personality and clinical data (e.g., TCI, SAPS and SANS), was calculated in the same fashion. Box plots were used to show the differences between SZ and BP patients at their original averaged correlation values for selected connections within a bicluster, and compare them to the same connections in controls, other BPs, and other SZs not included in the bicluster (Fig. S2). Paired t-test was used to assess the results of the box plot. Selection of significant correlations and redesign of box plots illustrate the predominant functional connections in the bicluster, sometimes only affecting BP patients or only SZ patients (Figs. S2T-U, S9F).

**(6) Graph and matrix representations of biclusters**

*(6.1) Displaying biclusters extracted after factorization (Figs. S2, S9).* Correlation values from a bicluster were visualized as a heat map, where each column represents a theme and each row represents a connection. An alternative representation includes graphs and their corresponding adjacent matrices. In a graphical representation, each node corresponds to a network (ie auditory, visual...) and each edge corresponds to the number of connections between network parcels represented as nodes. The blue and red borders represent correlation values above and below the control average for these connections, respectively. The underlying matrix constitutes an alternative representation and is used throughout the work.

*(6.2) Displaying and decoding TBSS biclusters and transforming them back to native space(Figs. 2, S1, S6).* Each structural bicluster was converted back to its native domain of knowledge producing plots for the corresponding axial, sagittal, and coronal views. For example, the NifTi files corresponding to a bicluster are combined into a new NifTi file that summarizes the FA voxels shared by its members. The average FA skeleton image was then loaded on top of the background image in FSLView and its display range was set appropriately, to show where the skeleton was estimated and which standard space voxels were tested in multi-subject statistics. Changed colormap to highlight results. The whole process can be seen at <http://web.mit.edu/fsl_v5.0.10/fsl/doc/wiki/TBSS(2f)UserGuide.html>

*(6.3) Displaying and decoding fMRI biclusters and transforming them back to native space (Figs. 3, S3, S6).* The BrainNet Viewer, a brain network visualization tool, was used and helped us to visualize functional connectivity patterns from different levels in a quick, easy, and flexible way. The nodes in Brainnet viewer were defined by Gordon parcellation in AAL90 atlas. This is an estimation of the center of the area corresponding to the network. We use the Brainnetviewer for Matlab based on <https://www.nitrc.org/projects/bnv/>.

**Notes on Connectivity Circuitry**

1) Amygdala network (AmN)

The AmN is an automatically activated network that is widely thought to form the core of a neural system for processing fearful and threatening stimuli, including detection of threat and activation of appropriate fear-related behaviors in response to threatening or dangerous stimuli. Self-regulation of activity in the amygdala can improve emotional perception and recognition as a novel neurofeedback training method. Disrupted functional connectivity of the amygdala may be important in mental disorders, especially those involving fear. Both rural living^39^ and nature walks^40^ are associated with reduced amygdala activity. Individual differences in TCI Harm Avoidance are positively correlated with amygdala activation in response to unpleasant or aversive stimuli, which is associated with activation of the Salience Network in collaboration with other prefrontal top-down networks (DMN, CON, FPN)^41-43^. The amygdala encodes and affects social emotional stimuli when processing faces and subjective judgments of facial expression, and it collaborates with many other regions of brain through the Perception Network to process this social emotional information.

2) Auditory Network (AuN)

The AuN is an automatically activated network that informs interaction with the environment which ranges from processing auditory information to other sensory and cognitive tasks, including tonal, pitch, speech, and language processing^44, 45^. The auditory cortex is the part of the temporal lobe that processes auditory information in humans and many other vertebrates. It is a part of the auditory system, performing basic and higher functions in hearing, such as possible relations to language switching.

3) Cingulo-Opercular Network (CON)

The CON is one of the four prefrontal top-down networks that collaborate in voluntary processes. It is comprised of brain regions including the anterior insula/operculum dorsal anterior cingulate cortex, and thalamus. Its function has been particularly difficult to characterize due to the network's pervasive activity and frequent co-activation with other control-related networks. It is one of two networks regulating executive top-down control and is distinguished by its role in sustained maintenance of intentional control, in contrast to the flexible and rapid executive control by the Fronto-Parietal network that allows rapid adaptation to task demands^46^. Consequently, it is variously described as regulating effortful control or effortful tonic alertness ^47^. It is also called the network for sustained or executive attention in contrast to the dorsal attention network for selective attention ^48, 49^. Effortful control is measured as a personality trait by Posner and his colleagues by a measure corresponding in the TCI to high Self-directedness, high Persistence, and low Harm Avoidance (i.e., SPh) ^50^.

4) Context Network (CN)

The CN involves functional communication among the medial prefrontal cortex, retrosplenial cortex, and parahippocampal cortex, which facilitates the recognition of objects in their typically associated context, including recognition of faces and objects that are expected in a particular context ^51^.

5) Default Mode Network (DMN)

The DMN is one of the four prefrontal networks that regulate collaborate in regulating top-down (voluntary) processes. It is active when a person is not focused on the outside world (i.e., when there is no external task to perform), as when a person is at rest or active with internal cognitive-emotional tasks requiring theory of mind and/or self-referential processing. It involves episodic memory retrieval, autobiographical memory, internal thought, self-rated and social cognitive processes, value-based decision making, emotion regulation, and mind wandering ^52^. It involves cognitive processes that are conducive to divergent thinking, whether realistic or unrealistic, including spontaneous imaginative thought, mind-wandering, perspective-taking, and imagining the future or recollecting the past, as reviewed elsewhere^53, 54^. Such divergent thinking is generated in collaboration with two other prefrontal networks, the Fronto-Parietal and Salience networks ^53, 54^. It is also enhanced by increased rsFC within the DMN in response to cognitive stimulation^55, 56^. The DMN includes medial prefrontal cortex, posterior cingulate cortex, ventral precuneus, medial temporal lobe, and parts of the parietal cortex.

6) Dorsal Attention Network (DAN)

The DAN is one of the four prefrontal networks that regulates top-down (voluntary, deliberate) processes. It is one of two attentional (i.e., sensory orienting) systems. It mediates top-down guided voluntary allocation to locations or features involving pursuit of goals. It focuses attention for consciously directed and rule-based problem solving and decision making that is goal-oriented ^57^, including working memory, planning and decision making. It does so in collaboration with the Fronto—Parietal Network (i.e., central executive network, mainly dorsolateral PFC and lateral posterior parietal cortex, especially supramarginal gyrus). It includes the intraparietal sulci and frontal eye fields. Consequently, highly self-directed people are expected to have increased activity of the DAN because it is hypothesized to be involved in generating and maintaining attentional sets by top-down cognitive control of stimuli and actions in a way that is voluntary and deliberate. It is comprised of the visual motion area, frontal eye fields, superior parietal lobule, intraparietal sulcus (IPS), and ventral premotor cortex.

7) Fronto-Parietal Network (FPN)

The FPN is one of the four prefrontal networks involved in top-down (voluntary, deliberate) processes. It regulates voluntary vigilance to adapt with rapid connectivity changes in response to brief events, usually as measured by reaction time to external cues. It provides phasic attention, including exogenously triggered initiation of control, adapting after errors, and moment-to-moment adjustment of control as in repeated task switching) ^47^. Its coupling shifts more than other regions when there is need for rapid switching of tasks, so is considered a functional hub for influencing brain-wide communication to meet task demands voluntarily. Consequently, it has a central role as a flexible hub for executive cognitive control and adaptive task control^58^, as demonstrated by rapidly changing its connectivity to mean task demands more variably than any other of the major cognitive brain networks^59^. In collaboration with the DMN and Salience Network, it plays a role in imaginative divergent thinking by selecting useful divergent ideas and inhibiting unoriginal ideas, as reviewed elsewhere^53, 54^. It involves the dorsolateral prefrontal cortex, middle frontal gyrus, and posterior parietal lobule, including intraparietal sulcus (IPS) or superior parietal lobule and the frontal eye fields^60^.

8) Entorhinal-Hippocampal Network (EHN)

The EHN is an automatically activated network that plays an essential role in episodic memory, which preserves spatial and temporal information about the occurrence of past events. Based on evidence from many fMRI connectivity studies, such hippocampal functioning is strongly linked to the DMN. It has been proposed that the DMN is a hippocampal memory network mediating episodic memory and other internally oriented operations, including thinking about the future and self-referential evaluation. People with damage to their hippocampus can recall memories prior to their brain damage but cannot create new ones. This is a phenomenon that leads some scholars to conclude that this brain structure serves as the gateway to all aspects of autobiographical memory, which is an aspect of the functioning of the DMN^61, 62^.

9) Perception Network (PerN)

The PerN is an automatically activated network that is important for social perceptual abilities. It is involved with the lateral orbitofrontal cortex, fusiform gyrus rostral superior temporal sulcus, ventromedial temporal cortex, ventral medial striatum of the nucleus accumbens, and others (see ^63^). The amygdala cooperates with many other regions of brain through the PerN to process social emotional information at a network level. The amygdala encodes and affects social emotional stimuli when processing faces and subjective judgments of facial expression.

10-11) Somatomotor networks for mouth (SM) and for hand (SH)

There are distinct Somatomotor networks for the mouth (SM) and hand (SH). They are automatically activated in collaboration with the top-down prefrontal networks. The SM network is a large-scale brain network that primarily includes somatosensory (postcentral gyrus) and motor (precentral gyrus) regions and extends to the supplementary motor areas (SMA)^64^. The auditory cortex may also be included. The primary somatosensory area occupies the postcentral gyrus, extending to the medial surface of the posterior part of the paracentral lobule. It receives input from the ventral posterior thalamic nucleus. Histologically there are three narrow strips of cortex (Brodmann areas 3, 1, 2); area 3, in the posterior wall of the central sulcus, has a granular heterotypical structure and responds to tactile stimuli; areas 1 and 2 have a homotypical structure and react to deep stimuli and joint movement. Nociceptive (pain or thermal) sensation reaches consciousness at thalamic level, but its qualitative and spatial evaluation is cortical. Gustatory impulses are received in the junctional region of the postcentral gyrus and insula. The supplementary somatosensory area II is in the superior lip of the lateral fissure, with the face anterior and the lower limb posterior (hand network). It has connections with the primary SM area (mouth network) and the thalamus, some being bilateral. Its functional significance is uncertain. Sensory and motor ‘speech areas’ exist only in one hemisphere, in the left hemisphere of right-handed individuals, and this is the ‘dominant hemisphere’. Damage results in word-blindness, alexia, and an inability to copy, agraphia, both being forms of sensory aphasia, the inability to understand written and spoken language.

12) Salience Network (SN)

The SN is one of the two bottom-up prefrontal networks and is primarily anchored by the anterior insula (AI) and dorsal Anterior cingulate cortex (dACC). The activity of the Salience network is positively correlated with TCI Harm Avoidance^42, 65-67^. In other words, the Salience network is associated with anxious avoidance of what is aversive, novel, and/or unfamiliar^68^. It detects and filters salient stimuli and recruits other functional networks thereby contributing to various complex functions, including communication, social behavior, and self-awareness by integration of sensory, emotional, and cognitive information. It is implicated in modulating the switch between the internally directed DMN and the externally directed cognition of the Cingulo-Opercular and FPN networks, so it functions collaboratively with these networks in harm-avoidant responses to negatively-valenced (aversive) signals^42^. The connectivity of the DMN with the insula in the Salience network is increased during the initial production of divergent thoughts^54^. In addition to AI and dACC, it includes substantia nigra, ventral tegmental area, ventral striatum, amygdala, dorsomedial thalamus, and hypothalamus. It is distinguished by von Economo neurons in the AI/dACC. The cortico-striatal-thalamic loop circuits contribute to the salience network.

13) Striatal Network (StN)

The STN is an automatically activated network with major involvement with the neocortex in behavioral conditioning of habits and skills. It involves the striate nucleus which serves as the primary input to the rest of the basal ganglia. Functionally, the striatum collaborates with prefrontal networks in the coordination of multiple aspects of cognition, including both motor and action planning, decision-making, motivation, reinforcement, and reward perception. The striatum is actively involved in neural computations needed for normal cognitive functions through its cortico-striato-cortical loops, so disconnection of this circuit is associated with positive symptoms in people with or at high risk for schizophrenia, including schizotypal personality disorder^11, 69, 70^.

14) Thalamus Network (TN)

The TN is an automatically activated network that encodes all information from body's senses except smell. This information must be processed through the thalamus before being sent to the brain cerebral cortex for interpretation. The thalamus also plays a role in sleep, wakefulness, consciousness, learning and memory. The thalamus is a mostly gray matter structure of the diencephalon that has many essential roles in human physiology. The thalamus is composed of different nuclei that each serve a unique role, ranging from relaying sensory and motor signals, as well as regulation of consciousness and alertness.

15) Ventral Attention Network (VAN)

The VAN is one of the two bottom-up prefrontal networks. It is involved in attention to external stimuli, such as reorienting or filtering attention toward stimuli in the environment that may be goal-directed. It detects novel or unexpected stimuli and triggers shifts of attention (task switching)^71^ in collaboration with other executive and attentional networks (FPN, DAN)^72^. It is lateralized to the right temporo-parietal junction with extension into the Inferior Parietal lobule (mainly angular gyrus) and the right ventro-frontal cortex. It is also described as a ventral fronto-temporo-parietal network for handing reflexive responses to exogenous stimuli or bottom-up reorienting, as when triggered by stimulus saliency and target detection, especially when they appear in novel or unexpected locations. It responds to detection of new behaviorally relevant stimuli outside the focus of goal-directed attention defined by the dorsal attention network. Its activity is increased after abrupt changes in sensory stimuli, at the onset and offset of task blocks, and at the end of a completed trial. In summary it is a stimulus-driven reorienting system that interrupts ongoing attention selection processes of the dorsal network when relevant stimuli occur.

16) Visual Network (VisN)

The VisN is specialized for processing information about static and moving objects. Its dorsal stream is associated with spatial awareness and guidance of actions, and the ventral stream is associated with object recognition ^73^. Vision is central to human experience. The neural substrate of vision is a network of cortical and subcortical brain areas, organized in partially segregated but interacting processing streams. Collectively, neurons across this network analyze the various components of the visual scene, which are then assembled into a coherent visual percept. Dysfunctions of the visual system range from scotomas, in which vision in part of the visual field is diminished or absent, to impairments of high-order visual functions that result in perplexing neurological symptoms such as deficiency in face recognition or an inability to distinguish between self- and externally generated motion.

**Clinical Profiles Distinguish each rsFC group**

Clinical characteristics were distinguished by significantly elevated SANS/SAPS global ratings for all the groups except group 3, as shown in Table 1. The individuals in group 3 did not show significant differences in any of the global symptom scores, but they had significantly higher scores on two more specific clinical features – thought blocking and somatic delusions (Table 1, Supplemental Table S1, S5, p < 6.83E-04).

The symptoms of negative self-regulatory functions that distinguished each of the five rsFC groups corresponded to what was expected from their temperament and character profiles, as described in the prior section (Table 1). When the four prefrontal self-regulatory networks were all highly connected, as in groups 1 and 2, so were their automatically co-activated networks. However, when one or more of the prefrontal self-regulatory networks were more weakly connected than controls, so were proportionate numbers of their automatically co-activated networks (Table 1).

Among the positive symptoms, verbal hallucinations and delusions were associated with reduced rsFC in the striatum and auditory regions involved in processing language, as expected for Fragile-Avolitional subjects (group 3). Somatic Delusions and blocking of the flow of thought were associated with Novelty Seeking (N) and hostile mistrust (cT) in subjects in group 4 whose automated modules were all positive except for negative somatomotor control of the mouth.

Among the disorganized positive symptoms, bizarre behavior and positive formal thought disorder were observed in the ambivalent-overattentive schizotypes (group 2) whose automatically co-activated modules all had higher rsFC than controls. In contrast, the inattentive schizotypes (group 5) had reduced rsFC in the auditory, context, perception, and somatomotor-hand modules.

**Detailed Relations of rsFC and other features within groups to Diagnoses**

There were some significant but weak differences in rsFC between SZ and BP subjects (see Supplementary Information) within the groups. In the Avoidant-Anhedonic subjects (group 1), only four out of its 279 network connectivity correlations (Table 2) differed significantly between patients with BP and SZ, with three higher in SZ (Supplementary Table S1, Figure S2D, 4.70E-07<p<4.68E-02.

The Sensitive-Disorganized subjects (group 2) had 12 of 1077 network connectivity correlations (Table 2) that distinguished BP from SZ patients with higher correlation values in those with SZ (Supplementary Table S1, Figure S2H, 1E-03<p<4.55E-14).

In the Asocial-Blocked subjects (group 3), the connectivity correlations were similar in pattern but significantly stronger in BP than SZ patients between the Visual (p<3.11E-03), Auditory (p<8.48E-05), and Thalamus (p<3.51E-03) networks with the DAN, VAN, Context, and Perception networks (Supplementary Tables S1, S2). Likewise, the correlations of the CON with the DAN, VAN, and Somatomotor networks were stronger in BP than SZ patients (Supplementary Figure S2L).

In the Fragile-Avolitional subjects (group 4), there were no significant differences among or within the connections of the prefrontal networks. Several weak connectivity correlations of the CON with other co-activated networks (including DAN (p<3.74E-03) and auditory (p<1.63E-02) networks differed between patients with SZ and BP, but equal numbers were relatively stronger for subjects with each diagnosis (Supplementary Tables S1, S2, Figure S2P).

The patients with BP and SZ in group 5 were highly similar in their rsFC. Among its 209 connections (Table 2), there was only one difference in intrahemispheric connectivity within the CON that distinguished them significantly (Supplementary Tables S1, S2, Figure S3S, p<1.5E-05).

In addition to SAPS/SANS ratings that distinguished the composite groups of patients, some additional clinical features distinguished BP and SZ patients (Supplementary Tables 2-6), but these were confounded with the diagnostic criteria. For example, patients with BP had more affective symptoms whereas patients with SZ had more frequent hallucinations (p<7.35E-05) and delusions (p<2.81E-06).

***Structural connectivity distinguishes each Functional Connectivity Group***

Decreased fractional anisotropy (FA, in red, p<0.05 after adjustment for multiple comparisons of voxels) maps of white matter microstructure distinguished the five groups of subjects that we identified based on their rsFC alone (Figure 4, Supplementary Table S1). The microstructural connectivity pattern for each group was distinct, but all of them had significantly asymmetric involvement, more severe in the left hemisphere. White matter tracts associated with the different rsFC groups primarily involved prefrontal fibers and their connections to motor, limbic, and cerebellar regions. For most components of each rsFC group, we observed corresponding white matter structural abnormalities, as shown in Supplementary Table S1 (see columns low FA and known structural connections).

Figure 4 about here

The Avoidant-Anhedonic individuals (group 1) (Figure 4A, Supplementary Table S1) had reduced FA in regions known to be related to the components of rsFC group, including the posterior and superior corona radiata, inferior cerebellar peduncle, and the anterior limb of the internal capsule. Only Avoidant-Anhedonic individuals were found to have low FA in their fornix, which connects the hippocampus with other nodes of the limbic system and thereby plays a key role in emotion regulation.

Sensitive-Disorganized patients (group 2) (Figure 4B, Supplementary Table S1) also shared reductions in FA in the superior corona radiata, inferior cerebellar peduncle, and middle cerebellar peduncle, which support the functioning of the prefrontal CON, DMN, DAN networks. This group was unique in its abnormalities in all three of these white matter regions.

Reduced FA in Asocial-Blocked patients (group 3) (Figure 4C, Supplementary Table S1) was also found in the anterior limb of internal capsule, superior and posterior corona radiata, as well as in the superior cerebellar peduncle and the body of the corpus callosum that support the functioning of its functional components. Low FA was only observed uniquely in the superior cerebellar peduncle of people in group 3, which is a component of the cortico-cerebellar-cortical loop that is important for the function of the Default Mode.

Reduced FA in Fragile-Avolitional patients (group 4) (Figure 4D, Supplementary Table S1) was found in many fibers of the prefrontal networks. These included cortical association fibers of the inferior and superior fronto-occipital fasciculi, the inferior longitudinal fasciculus, and the external capsule, as well as the frontal-thalamic and frontal-striatal connections within the anterior corona radiata, as well as in the afferent and efferent fibers of the primary motor and somatosensory cortex, auditory and visual input in the posterior limb of internal capsule, and the superior cerebellar peduncle. The Fragile-Avolitional group was unique in having low FA in the thalamic radiation to the posterior limb of the internal capsule, which supports the functioning of the executive attention network (CON).

Lastly, Explosive-Inattentive individuals (group 5) (Figure 4E) shared decreases in FA in the anterior limb of internal capsule, cortico-cortical association fibers in the superior longitudinal fasciculus, cingulum and related cingulate cortex, and interhemispheric frontal connections in the genu of corpus callosum. In addition, they shared decreased FA in the visual afferents in the posterior thalamic radiations, and in the inferior, middle, and superior cerebellar peduncles, including the decussation. Explosive-Inattentive individuals were unique in having low FA in the cingulum and superior longitudinal fasciculus, white matter tracts of the DMN, CON, and DAN that connect frontal, temporal, and parietal cortices.

There were few differences in FA according to diagnosis within the groups. Four groups showed excess FA in particular voxel-sparse regions, but only in the BP patients (Figure 4). The Avoidant-Anhedonic individuals comprised the only group with excess FA in both BP and SZ patients, and the excess FA in BP patients were in different brain regions than that found in SZ patients (Figure 4).

**Collaborative relations of prefrontal and automatically-coactivated networks**

The hypothesis that the other automatically-coactivated networks function collaboratively with the prefrontal networks was well supported, as summarized in Table 1 of the main article. For example, the Asocial-Blocked subjects (group 3) had increased rsFC in most other automatic networks including those involved in social perception (Context Network, Perception Network), communication (Auditory/language processing) along with reduced connectivity in the somatomotor network for the mouth (Table 1). The Fragile-Avolitional subjects (group 4) had reduced connectivity in the Cingulo-Opercular, Somatomotor (hand and mouth), Striatum, Auditory and Visual networks (Table 1, Supplementary Table 1). Other networks for evaluation of evaluation of faces and social emotions (Context and PerN) had increased connectivity in hostile mistrustful subjects (group 3) and decreased activity in inattentive schizotypes (group 5).

**Supplementary Table and Figure Legends**

**Table S1**. Structural, personality, and clinical features that distinguish the five functional connectivity groups.

Functional network components (fMRI) are listed alongside their known structural connections. Highly connected fMRI components are shown in blue. Distinguishing clinical features are shown for TCI, WERCAP, and SAPS/SANS for all cases per group together and by diagnoses of BP or SZ. Differences between SZ and BP are shown, including whether network components were more active in patients with BP or SZ (indicated by diagnosis in parenthesis). Red values in the TCI indicate significant differences from controls.

**Table S2**. Significant differences between BP and SZ functional connectivity. High Correlation indicates which disease displays higher values and % shows similar interactions but distinct higher correlation disease.

**Table S3**. Dissection of functional connectivity into personality profiles. Results from all SZ+BP, BP, and SZ within each connectivity group.

**Table S4**. Dissection of the functional connectivity into Global SAPS and SANS categories. Results from all SZ+BP, BP, and SZ within each connectivity group.

**Table S5**. Specific clinical phenotypes. Results from all SZ+BP, BP, and SZ within each connectivity group. Significant values were highlighted in blue.

**Table S6**. Dissection of the functional connectivity into global clinical symptoms. ANOVA Statistics indicates groups vs "others", where "others - controls" or "others - BP -SZ”. Results from all SZ+BP, BP, and SZ within each connectivity group.

**Table S7**. Comparison of demographic characteristics, duration of illness, and psychotropic medication history in uncovered rsFC groups of patients (groups 1-5) and controls.

Notes: Handedness (right or left). Psychotropic medications include typical and atypical neuroleptics, other mood stabilizers (carbamazepine, divalproex, oxcarbazepine, topirimate, lamotrigine), other antidepressants (SSRIs). Duration of illness is in months. Statistical tests of inter-group differences are summarized here and detailed in Table S8. Percentage of pairwise comparisons with no significant difference (p >0.05) or weak effect size (r <0.3) are summarized here.

**Table S8:** Comparison of controls and patient groups for demographics, duration of illness, and treatment history.

Statistical results are shown using chi-square test for categorical variables and using ANOVA and t-tests for categorical vs numerical variables (Matlab, Statistical Toolbox). The source of the variability is between groups. Chi-square H = 1 suggests different distributions. P is the corresponding p-value for rejecting the null hypothesis. Test indicates the value of the statistical test. Df indicates the degrees of freedom, which is one for Chi-square tests, and varies for ANOVA tests. In the ANOVA tests, the Sum of squares (SS) due to each source, and Mean Squares (MS) for each source, which is the ratio SS/df are reported. Effect size was determined by the correlation coefficient, properly accounting for the size of the groups and any overlap in group memberships. Note that Handedness is right or left.

Psychotropic medications include typical and atypical neuroleptics, other mood stabilizers, lithium, SSRI/SNRI antidepressants, other antidepressants, benzodiazepines, and anticholinergics. Duration of illness is in months.

|  |  |  |  |  |  |  |  |  |  |  |  |  |  |  |  |  |  |  |  |  |  |  |  |  |  |  |  |  |  |  |  |  |
| --- | --- | --- | --- | --- | --- | --- | --- | --- | --- | --- | --- | --- | --- | --- | --- | --- | --- | --- | --- | --- | --- | --- | --- | --- | --- | --- | --- | --- | --- | --- | --- | --- |

**Figure S1**. Graph representation of to the five fMRI connectivity groups using BrainNet Viewer (complementary to Figure 3).

Thicker edges correspond to stronger correlations (only the most notorious edges are shown). Inter-hemisphere links encode correlations within each functional network. (A-E) represent each of the five sets that describe different patterns of connections among the nodes: (A)The Avoidant-Anhedonic (group 1), (B) the Sensitive-Disorganized subjects (group 2), (C) the Asocial-Blocked subjects (group 3), (D) the Fragile-Avolitional subjects (group 4), and (E) the Explosive-Inattentive individuals (group 5).

**Figure S2**. Series of four comparable figures for each group

Bicluster; graph diagram; Boxplot of the set, which splits SZ and BP patients vs. Others, and SZ or BP or CNT vs others). Boxplot shows similarities and differences between SZ and BP patients at the sub-network levels for fMRI group 1 (A-D), group 2 (E-H), group 3 (E-H), group 4 (I-L), for group 5 (M-P). (U) Shows a boxplot like that of (T) after selecting the differential subnetworks from (S).

**Figure S3**. TBSS high FA images corresponding to SZ and BP subjects of each group (from top to bottom).

(A-E) represent each of the five sets that describe different patterns of connections among the nodes: (A) The Avoidant-Anhedonic (group 1), (B) the Sensitive-Disorganized subjects (group 2), (C) the Asocial-Blocked subjects (group 3), (D) the Fragile-Avolitional subjects (group 4), and (E) the Explosive-Inattentive individuals (group 5). The first and second lines of each group correspond to BP and SZ patients in the groups, respectively.

**Figure S4.** Comparisons between significant fMRI sets and more specific sets which in addition to being significant, show preponderance of either SZ or BP (Table S1).

Four sets that describe different patterns of connections among the nodes are illustrated: (A) the Sensitive-Disorganized subjects (group 2), (B)The Avoidant-Anhedonic (group 1), (C) the Asocial-Blocked subjects (group 3), and (D) the Fragile-Avolitional subjects (group 4).

**Figure S5**. Characterization of methods for uncovering groups.

Generic data (red dots), clustering partitions (circle, ovals, rectangles), and their membership scopes as defined by their most characteristic distance metrics are represented. (A) Model‑based clustering is applied to linearly distributed datasets, where the most appropriate prototype (centroid) is a line rather than a single point. Thus, there is a single instead of three clusters. (B) Fuzzy clustering (e.g., C‑means) applied to vaguely distributed datasets. (C) Possibilistic clustering applied to datasets with several outliers. (D) Feature selection included in the clustering process (e.g., biclusters) applied to datasets with patterns involving different sets of features. (E) Consensus clustering is applied to datasets that can be correctly interpreted as rectangles and/or triangles (left panel) accordingly to the maximum number of clusters being considered (*k*=2 or *k*>2). Two clusters (rectangles) are appropriately identified with *k*=2 (upper panel), whereas only one partition is meaningful with *k*=4 (left panel) and another is superfluous and unrequired (right panel, dashed lines). (F) Example of an object (circle, Conceptual clustering) uncovered at different levels of sensitivity (outer circle) and specificity (inner circle).

**Figure S6**. The matrix factorization process is depicted as a deep unsupervised learning approach, where data are pre-processed, encoded into a flattened vector, processed as a convolutional network (NMF) and decoded into representative patterns of each data group.

**Figure S7**. Schematic of the machine learning method. (A) Deep unsupervised NMF learning process: (i) NMF is implemented based on decomposing an input dataset, encoded as a matrix (or a tensor) composed of features and observations/subjects, into smaller factors. The learning process is a mirror process because it consists of comparing the original matrix with that reconstructed from the factors and adjustments of those factors by the error. Factors are derived by combining matrix W and H. (iii) NMF can be transformed into a supervised method by moving matrix W to the other side of the equation applying the pseudoinverse of a product. (iii) Illustration of the process carried out by the NMF method to learn one factor: ordering the columns of W, as well as the rows of H, and multiplying them. (B) Deep NMF process systematically applied (convolutive) using different number of maximum clusters or granularity levels (Consensus clustering). Optimal submatrices (factors) are selected from all levels by a multi-objective optimization process. This image can also illustrate the recurrent application of NMF to identify higher-level profiles. (C) Schematic that exemplifies how NMF sees the biomedical datasets (GWAS, DTI images, etc.). 6 patients have a deficit (value = 20) in different regions. Typically, the average of each cell is calculated, and, consequently, there is no region with a particular deficit (all values = 87) in all patients. Because averaging the cells conceals the differences among patients, NMF is focused on segmentation of patients and features into distinct groups defined by subgroups of subjects with particular features.

**Figure S8**. Graphs and clusters. (A) An adjacency matrix is a square matrix used to represent a finite graph. The elements of the matrix indicate whether pairs of vertices are adjacent or not in the graph. Every matrix has its corresponding graph and vice versa. (B) A bipartite graph (or bigraph) is a graph whose vertices can be divided into two disjoint and independent sets U and V, that is every edge connects a vertex in U to one in V. (C) A clique is a subset of vertices of an undirected graph such that every two distinct vertices in the clique are adjacent. A graph with 23 × 1-vertex cliques (the vertices), 42 × 2-vertex cliques (the edges), 19 × 3-vertex cliques (light and dark blue triangles), and 2 × 4-vertex cliques (dark blue areas). The 11 light blue triangles form maximal cliques. The two dark blue 4-cliques are both maximum and maximal, and the clique number of the graph is 4. (D) A bicluster is a bipartite graph with maximal cliques. A bipartite graph G1 has an edge maximum biclique B1({u1, u2},{v1,v2,v3}) with 5 vertices and 6 edges, and a vertex maximum biclique B2({u3,u4,u5,u6,u7},^74^) with 6 vertices and 5 edges. Both B1 and B2 are maximal. V and U may represent subjects and features, respectively. Methods that pre-impose a specific number of clusters, as well as a full and crisp membership of the observations to them, add edges where they do not exist, so they are biologically unrealistic.

**Figure S9**. Functional connectivity representations. (A) a graph of connections selected for each bicluster. Each node corresponds to a network (i.e., Auditory, Visual, etc.), whereas each edge corresponds to the number of connections observed in the evaluated subjects between network parcel nodes. Blue edges represent correlation value above the control average, and red edges represent correlation value under control average. The image encode these connections: High -- Visual_1 -- Visual_3, High -- Visual_4 -- Visual_8, Low -- Auditory_7 -- Auditory_10, High -- Visual_2 -- Auditory_9 and High -- Visual_12 -- Auditory_6. (B-C) Matrix summarizing graph representation of functional connections. Cells represents graph edges. (D) 17 networks summarize 369 sub-network connections. (E) Matrix factorization and posterior dissection into biclusters are represented as heatmap reconstructing the original correlation values. Each column represents a subject, and each row represents a connection (Auditory Vs. Visual, etc.). (F) Boxplot showing statistical significance of SZ and BP connections within a bicluster.

**Figure S10**. Selection of biclusters. (A) Representation of the algorithm used to select biclusters based on multi-objective and niching optimization techniques. (B) Niches calculated based on the Jaccard’s Index applied to (B, D) subjects or (C, E) features shared among three and four selected biclusters. As a consequence, none of these biclusters compete with each other and were selected.

**Figure S11**. Recurrent occurrence of the five biclusters organized into niches by similarity (Jaccard’s Index, which varies from 0 to 1 or 0% to 100%). (A-E) represent each of the five sets that describe different niches of each group: (A) The Avoidant-Anhedonic (group 1), (B) the Sensitive-Disorganized subjects (group 2), (C) the Asocial-Blocked subjects (group 3), (D) the Fragile-Avolitional subjects (group 4), and (E) the Explosive-Inattentive individuals (group 5).

**References**

1. Arnedo J, Mamah D, Baranger DA, Harms MP, Barch DM, Svrakic DM *et al.* Decomposition of brain diffusion imaging data uncovers latent schizophrenias with distinct patterns of white matter anisotropy. *Neuroimage* 2015; **120:** 43-54.

2. Zwir I, Zaliz RR, Ruspini EH. Automated biological sequence description by genetic multiobjective generalized clustering. *Ann N Y Acad Sci* 2002; **980:** 65-82.

3. Cordon O, Herrera F, Zwir I. Linguistic modeling by hierarchical systems of linguistic rules. *Ieee T Fuzzy Syst* 2002; **10**(1)**:** 2-20.

4. Zwir I, Shin D, Kato A, Nishino K, Latifi T, Solomon F *et al.* Dissecting the PhoP regulatory network of Escherichia coli and Salmonella enterica. *Proc Natl Acad Sci U S A* 2005; **102**(8)**:** 2862-2867.

5. Romero-Zaliz R, C. Rubio R, Cordón O, Cobb P, Herrera F, Zwir I. A multi-objective evolutionary conceptual clustering methodology for gene annotation within structural databases: a case of study on the gene ontology database. *IEEE Transactions on Evolutionary Computation*  2008; **12:6:** 679-701.

6. Harari O, Park SY, Huang H, Groisman EA, Zwir I. Defining the plasticity of transcription factor binding sites by Deconstructing DNA consensus sequences: the PhoP-binding sites among gamma/enterobacteria. *PLoS computational biology* 2010; **6**(7)**:** e1000862.

7. Arnedo J, del Val C, de Erausquin GA, Romero-Zaliz R, Svrakic D, Cloninger CR *et al.* PGMRA : a web server for ( phenotype x genotype ) many-to-many relation analysis in GWAS. *Nucleic Acid Research* 2013; **75**(Web Server Issue).

8. Arnedo J, Svrakic DM, del Val C, Romero‑Zaliz R, Hernández-Cuervo H, Molecular Genetics of Schizophrenia Consortium *et al.* Uncovering the Hidden Risk Architecture of the Schizophrenias: Confirmation in Three Independent Genome-‑Wide Association Studies. *American J of Psychiatry* 2015; **172**(2)**:** 139-153.

9. Zwir I, Arnedo J, Del-Val C, Pulkki-Raback L, Konte B, Yang SS *et al.* Uncovering the complex genetics of human temperament. *Mol Psychiatry* 2018.

10. Zwir I, Del-Val C, Arnedo J, Pulkki-Raback L, Konte B, Yang SS *et al.* Three genetic-environmental networks for human personality. *Mol Psychiatry* 2019.

11. Zwir I, Del-Val C, Hintsanen M, Cloninger KM, Romero-Zaliz R, Mesa A *et al.* Evolution of genetic networks for human creativity. *Mol Psychiatry* 2022; **27**(1)**:** 354-376.

12. Automated Qualitative Description of Measurements. *Proceedings of the Proceedings of the 16th IEEE Instrumentation and Measurement Technology Conf.*1999; Venice, Italy.

13. Ruspini EH, Zwir I. Automated generation of qualitative representations of complex objects by hybrid soft-computing methods. In: Pal SK, Pal A (eds). *Pattern recognition : from classical to modern approaches*. World Scientific: New Jersey., 2002, pp 454-474.

14. Bezdek JC, Pal SK, IEEE Neural Networks Council. *Fuzzy models for pattern recognition : methods that search for structures in data*. IEEE Press: New York, 1992, xi, 539pp.

15. Fred AL, Jain AK. Combining multiple clusterings using evidence accumulation. *Pattern Analysis and Machine Intelligence, IEEE Transactions on* 2005; **27**(6)**:** 835-850.

16. Latorre Carmona P, Sánchez JS, Fred ALN, SpringerLink (Online service). Mathematical Methodologies in Pattern Recognition and Machine Learning Contributions from the International Conference on Pattern Recognition Applications and Methods, 2012. *Springer Proceedings in Mathematics & Statistics,*. Springer New York : Imprint: Springer,: New York, NY, 2013, pp VIII, 194 p. 158 illus., 140 illus. in color.

17. Bittner T, Smith B. A theory of granular partitions. *Foundations of geographic information science* 2003**:** 117-151.

18. Fraley C, Raftery AE. How many clusters? Which clustering method? Answers via model-based cluster analysis. *The computer journal* 1998; **41**(8)**:** 578-588.

19. Senbabaoglu Y, Michailidis G, Li JZ. Critical limitations of consensus clustering in class discovery. *Sci Rep* 2014; **4:** 6207.

20. Saeed F, Salim N, Abdo A. Voting-based consensus clustering for combining multiple clusterings of chemical structures. *J Cheminform* 2012; **4**(1)**:** 37.

21. Zwir I, Huang H, Groisman EA. Analysis of differentially-regulated genes within a regulatory network by GPS genome navigation. *Bioinformatics* 2005; **21**(22)**:** 4073-4083.

22. Bezdek JC. Pattern Analysis. In: Pedrycz W, Bonissone PP, Ruspini EH (eds). *Handbook of Fuzzy Computation*. Institute of Physics: Bristol, 1998, pp F6.1.1-F6.6.20.

23. Kim EY, Kim SY, Ashlock D, Nam D. MULTI-K: accurate classification of microarray subtypes using ensemble k-means clustering. *BMC bioinformatics* 2009; **10:** 260.

24. Zhang Y, Phillips CA, Rogers GL, Baker EJ, Chesler EJ, Langston MA. On finding bicliques in bipartite graphs: a novel algorithm and its application to the integration of diverse biological data types. *BMC bioinformatics* 2014; **15:** 110.

25. Cichocki A. *Nonnegative matrix and tensor factorizations : applications to exploratory multi-way data analysis and blind source separation*. John Wiley: Chichester, U.K., 2009, xxi, 477 p.pp.

26. Deb K. Nonlinear goal programming using multi-objective genetic algorithms. *J Oper Res Soc* 2001; **52**(3)**:** 291-302.

27. Deb K. *Multi-objective optimization using evolutionary algorithms*. 1st edn. John Wiley & Sons: Chichester ; New York, 2001, xix, 497pp.

28. Gordon EM, Laumann TO, Adeyemo B, Huckins JF, Kelley WM, Petersen SE. Generation and Evaluation of a Cortical Area Parcellation from Resting-State Correlations. *Cerebral Cortex* 2014; **26**(1)**:** 288-303.

29. Lee DD, Seung HS. Learning the parts of objects by non-negative matrix factorization. *Nature* 1999; **401**(6755)**:** 788-791.

30. Pascual-Montano A, Carazo JM, Kochi K, Lehmann D, Pascual-Marqui RD. Nonsmooth nonnegative matrix factorization (nsNMF). *IEEE transactions on pattern analysis and machine intelligence* 2006; **28:** 403-415.

31. Tamayo P, Scanfeld D, Ebert BL, Gillette MA, Roberts CW, Mesirov JP. Metagene projection for cross-platform, cross-species characterization of global transcriptional states. *Proc Natl Acad Sci U S A* 2007; **104**(14)**:** 5959-5964.

32. Mejia-Roa E, Carmona-Saez P, Nogales R, Vicente C, Vazquez M, Yang XY *et al.* bioNMF: a web-based tool for nonnegative matrix factorization in biology. *Nucleic Acids Res* 2008; **36**(Web Server issue)**:** W523-528.

33. Brunet JP, Tamayo P, Golub TR, Mesirov JP. Metagenes and molecular pattern discovery using matrix factorization. *Proc Natl Acad Sci U S A* 2004; **101**(12)**:** 4164-4169.

34. Chagoyen M, Carmona-Saez P, Gil C, Carazo JM, Pascual-Montano A. A literature-based similarity metric for biological processes. *BMC bioinformatics* 2006; **7:** 363.

35. Chagoyen M, Carmona-Saez P, Shatkay H, Carazo JM, Pascual-Montano A. Discovering semantic features in the literature: a foundation for building functional associations. *BMC bioinformatics* 2006; **7:** 41.

36. Rissanen J. *Stochastic complexity in statistical inquiry*. World Scientific: Singapore, 1989, 177pp.

37. Romero-Zaliz R, Del Val C, Cobb JP, Zwir I. Onto-CC: a web server for identifying Gene Ontology conceptual clusters. *Nucleic Acids Res* 2008; **36**(Web Server issue)**:** W352-357.

38. Nguyen ND, Wang D. Multiview learning for understanding functional multiomics. *PLoS computational biology* 2020; **16**(4)**:** e1007677.

39. Zwir I, Arnedo J, Del-Val C, Pulkki-Råback L, Konte B, Yang SS *et al.* Three genetic-environmental networks for human personality. *Molecular Psychiatry* 2021; **26**(8)**:** 3858-3875.

40. Sudimac S, Sale V, Kuhn S. How nature nurtures: Amygdala activity decreases as the result of a one-hour walk in nature. *Mol Psychiatry* 2022.

41. Van Schuerbeek P, Baeken C, Luypaert R, De Raedt R, De Mey J. Does the amygdala response correlate with the personality trait 'harm avoidance' while evaluating emotional stimuli explicitly? *Behav Brain Funct* 2014; **10:** 18.

42. Baeken C, Marinazzo D, Van Schuerbeek P, Wu GR, De Mey J, Luypaert R *et al.* Left and right amygdala - mediofrontal cortical functional connectivity is differentially modulated by harm avoidance. *PLoS One* 2014; **9**(4)**:** e95740.

43. Van Schuerbeek P, Baeken C, De Raedt R, De Mey J, Luypaert R. Individual differences in local gray and white matter volumes reflect differences in temperament and character: a voxel-based morphometry study in healthy young females. *Brain Research* 2011; **1371:** 32-42.

44. Morillon B, Liégeois-Chauvel C, Arnal LH, Bénar CG, Giraud AL. Asymmetric function of theta and gamma activity in syllable processing: an intra-cortical study. *Frontiers in psychology* 2012; **3:** 248.

45. Zatorre RJ, Gandour JT. Neural specializations for speech and pitch: moving beyond the dichotomies. *Philos Trans R Soc Lond B Biol Sci* 2008; **363**(1493)**:** 1087-1104.

46. Dosenbach NU, Fair DA, Cohen AL, Schlaggar BL, Petersen SE. A dual-networks architecture of top-down control. *Trends Cogn Sci* 2008; **12**(3)**:** 99-105.

47. Sadaghiani S, D'Esposito M. Functional Characterization of the Cingulo-Opercular Network in the Maintenance of Tonic Alertness. *Cerebral cortex (New York, NY : 1991)* 2015; **25**(9)**:** 2763-2773.

48. Rueda MR, Checa P, Combita LM. Enhanced efficiency of the executive attention network after training in preschool children: immediate changes and effects after two months. *Dev Cogn Neurosci* 2012; **2 Suppl 1:** S192-204.

49. Rueda MR, Rothbart MK, McCandliss BD, Saccomanno L, Posner MI. Training, maturation, and genetic influences on the development of executive attention. *Proc Natl Acad Sci U S A* 2005; **102**(41)**:** 14931-14936.

50. Cloninger CR, Cloninger KM, Zwir I, Keltikangas-Jarvinen L. The complex genetics and biology of human temperament: a review of traditional concepts in relation to new molecular findings. *Transl Psychiatry* 2019; **9**(1)**:** 290.

51. Kveraga K, Ghuman AS, Kassam KS, Aminoff EA, Hämäläinen MS, Chaumon M *et al.* Early onset of neural synchronization in the contextual associations network. *Proc Natl Acad Sci U S A* 2011; **108**(8)**:** 3389-3394.

52. Raffone A, Marzetti L, Del Gratta C, Perrucci MG, Romani GL, Pizzella V. Toward a brain theory of meditation. *Progress in brain research* 2019; **244:** 207-232.

53. Zhu W, Chen Q, Xia L, Beaty RE, Yang W, Tian F *et al.* Common and distinct brain networks underlying verbal and visual creativity. *Human Brain Mapping* 2017; **38**(4)**:** 2094-2111.

54. Beaty RE, Benedek M, Kaufman SB, Silvia PJ. Default and Executive Network Coupling Supports Creative Idea Production. *Sci Rep* 2015; **5:** 10964.

55. Wei D, Yang J, Li W, Wang K, Zhang Q, Qiu J. Increased resting functional connectivity of the medial prefrontal cortex in creativity by means of cognitive stimulation. *Cortex* 2014; **51:** 92-102.

56. Takeuchi H, Taki Y, Hashizume H, Sassa Y, Nagase T, Nouchi R *et al.* The association between resting functional connectivity and creativity. *Cerebral cortex (New York, NY : 1991)* 2012; **22**(12)**:** 2921-2929.

57. Fox MD, Corbetta M, Snyder AZ, Vincent JL, Raichle ME. Spontaneous neuronal activity distinguishes human dorsal and ventral attention systems. *Proc Natl Acad Sci U S A* 2006; **103**(26)**:** 10046-10051.

58. Cole MW, Reynolds JR, Power JD, Repovs G, Anticevic A, Braver TS. Multi-task connectivity reveals flexible hubs for adaptive task control. *Nat Neurosci* 2013; **16**(9)**:** 1348-1355.

59. Zanto TP, Gazzaley A. Fronto-parietal network: flexible hub of cognitive control. *Trends Cogn Sci* 2013; **17**(12)**:** 602-603.

60. Hickok G, Small SL. *Neurobiology of language*. Elsevier/AP, Academic Press is an imprint of Elsevier: Amsterdam ; Boston, 2016, xxvii, 1159 pagespp.

61. Levine B. Autobiographical memory and the self in time: brain lesion effects, functional neuroanatomy, and lifespan development. *Brain and Cognition* 2004; **55**(1)**:** 54-68.

62. Spreng RN, Mar RA, Kim AS. The common neural basis of autobiographical memory, prospection, navigation, theory of mind, and the default mode: a quantitative meta-analysis. *J Cogn Neurosci* 2009; **21**(3)**:** 489-510.

63. Jiang Y, Tian Y, Wang Z. Causal Interactions in Human Amygdala Cortical Networks across the Lifespan. *Sci Rep* 2019; **9**(1)**:** 5927.

64. <https://www.sciencedirect.com/topics/neuroscience/superior-parietal-lobule>.

65. Paulus MP, Rogalsky C, Simmons A, Feinstein JS, Stein MB. Increased activation in the right insula during risk-taking decision making is related to harm avoidance and neuroticism. *Neuroimage* 2003; **19**(4)**:** 1439-1448.

66. Markett S, Weber B, Voigt G, Montag C, Felten A, Elger C *et al.* Intrinsic connectivity networks and personality: the temperament dimension harm avoidance moderates functional connectivity in the resting brain. *Neuroscience* 2013; **240:** 98-105.

67. Zwir I, Arnedo J, Del-Val C, Pulkki-Raback L, Konte B, Yang SS *et al.* Uncovering the complex genetics of human temperament. *Mol Psychiatry* 2020; **25**(10)**:** 2275-2294.

68. Williams LM. Precision psychiatry: a neural circuit taxonomy for depression and anxiety. *Lancet Psychiatry* 2016; **3**(5)**:** 472-480.

69. Waltmann M, O'Daly O, Egerton A, McMullen K, Kumari V, Barker GJ *et al.* Multi-echo fMRI, resting-state connectivity, and high psychometric schizotypy. *Neuroimage Clin* 2019; **21:** 101603.

70. Calvo de Padilla M, Padilla E, Gonzalez Aleman G, Bourdieu M, Guerrero G, Strejilevich S *et al.* Temperament traits associated with risk of schizophrenia in an indigenous population of Argentina. *Schizophr Res* 2006; **83**(2-3)**:** 299-302.

71. Vossel S, Geng JJ, Fink GR. Dorsal and ventral attention systems: distinct neural circuits but collaborative roles. *Neuroscientist* 2014; **20**(2)**:** 150-159.

72. Reineberg AE, Gustavson DE, Benca C, Banich MT, Friedman NP. The Relationship Between Resting State Network Connectivity and Individual Differences in Executive Functions. *Frontiers in psychology* 2018; **9:** 1600.

73. Nassi JJ, Callaway EM. Parallel processing strategies of the primate visual system. *Nature reviews Neuroscience* 2009; **10**(5)**:** 360-372.
