# Supplementary material for: Temperament & Character account for brain functional connectivity at rest: A diathesis-stress model of functional dysregulation in psychosis
Source: Mol Psychiatry. 2023 Apr 4;28(6):2238–53. doi: 10.1038/s41380-023-02039-6 (PMC10611583; doi:10.1038/s41380-023-02039-6)
Supplement: Supplementary file 2 — Supplementary Figures S1-S11 [file 41380_2023_2039_MOESM2_ESM.pptx]

## Slide 1
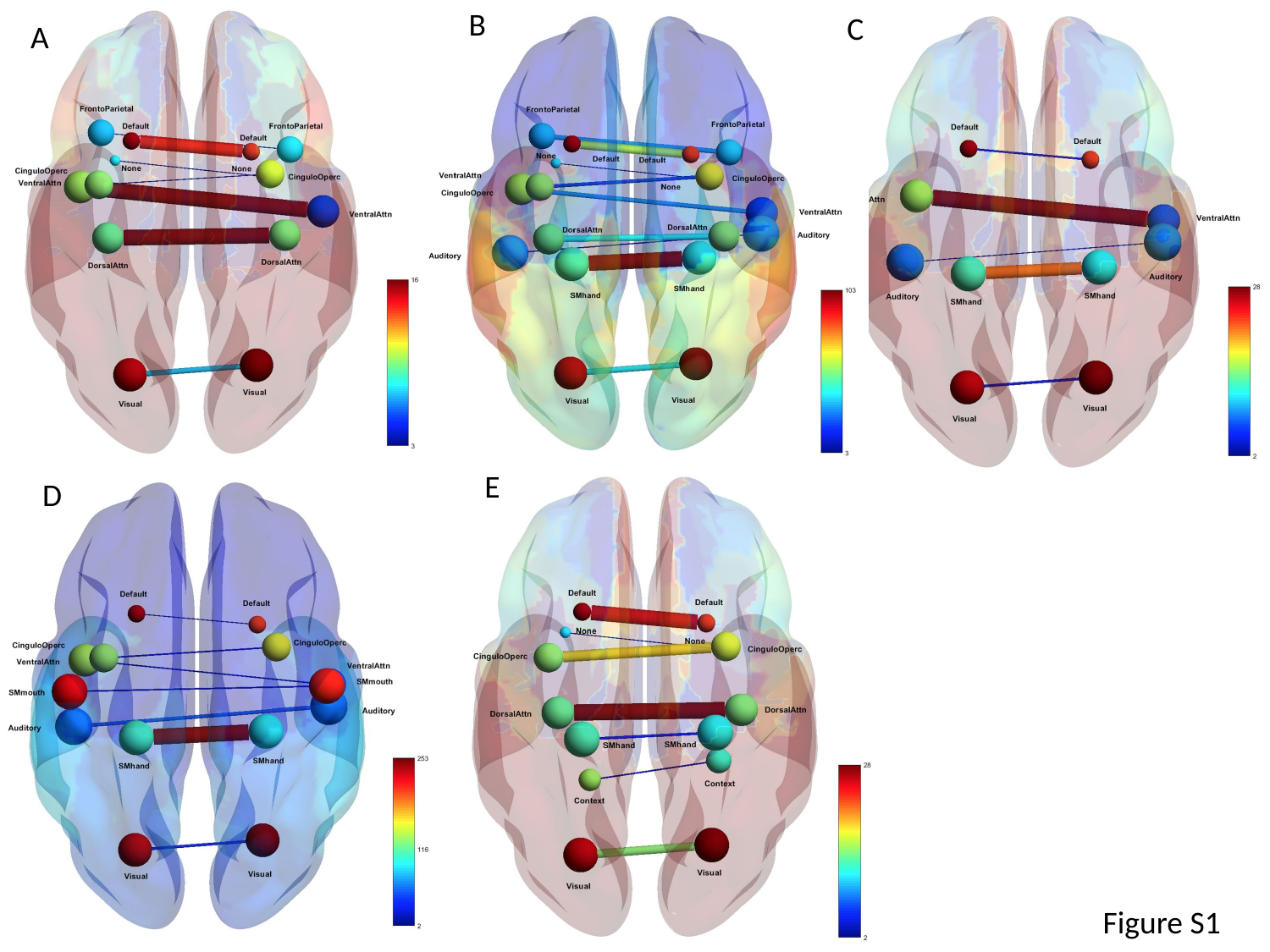

B
C
A
D
E
D
Figure S1

## Slide 2
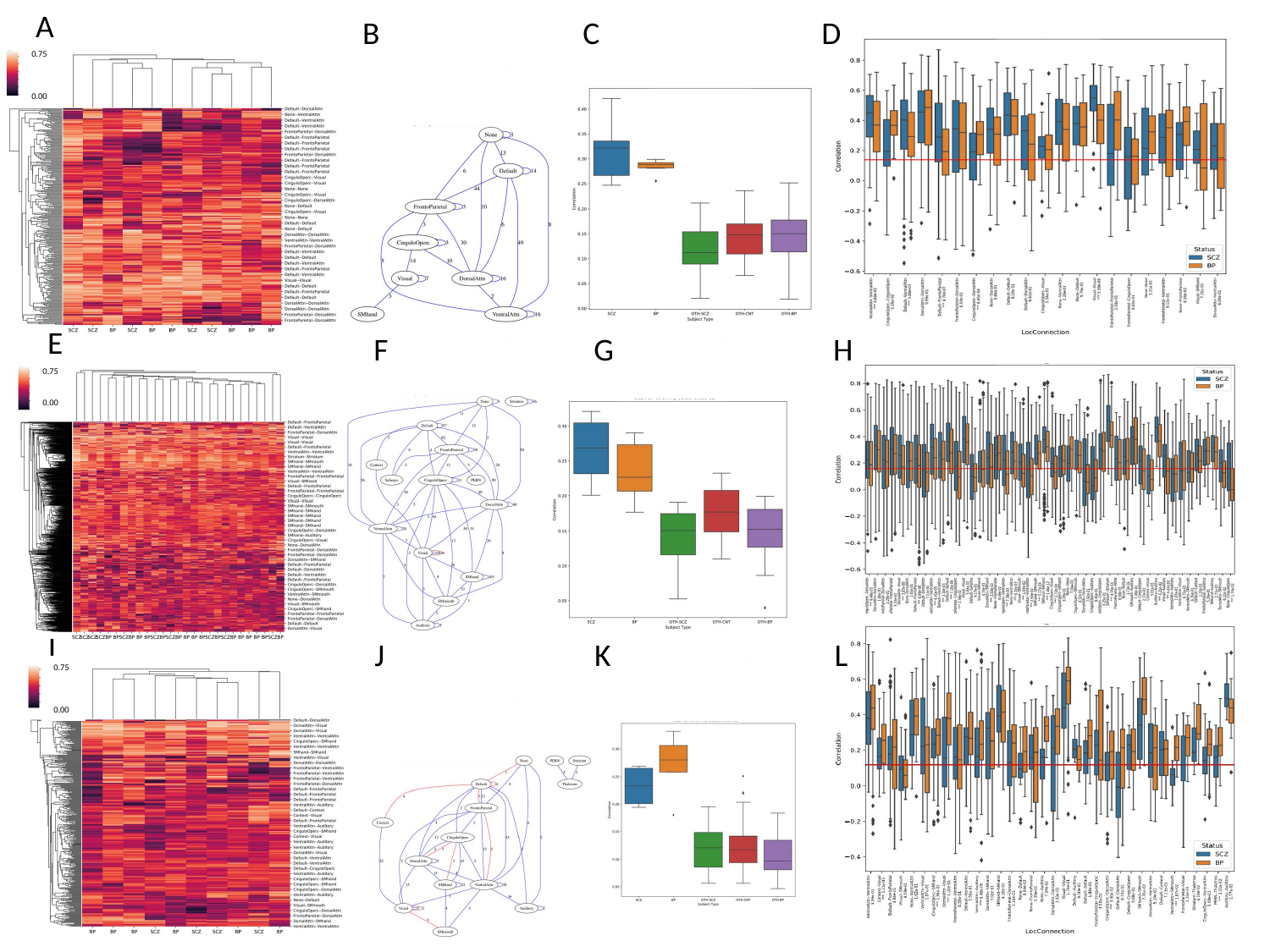

A
B
C
D
0.75
0.00
E
F
G
H
0.75
0.00
I
J
K
L
0.75
0.00

## Slide 3
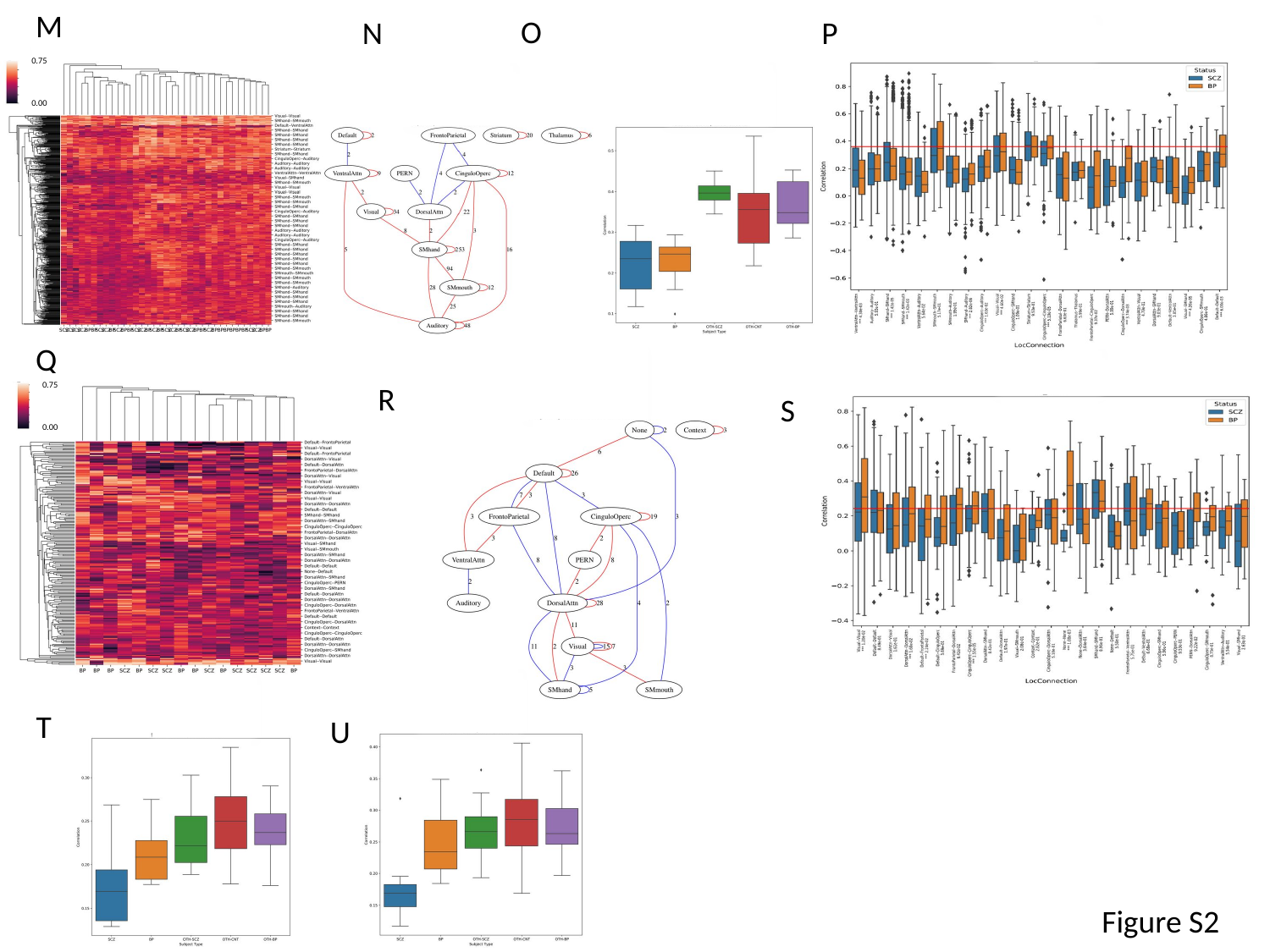

M
O
N
P
0.75
0.00
Q
0.75
0.00
R
S
T
U
Figure S2

## Slide 4
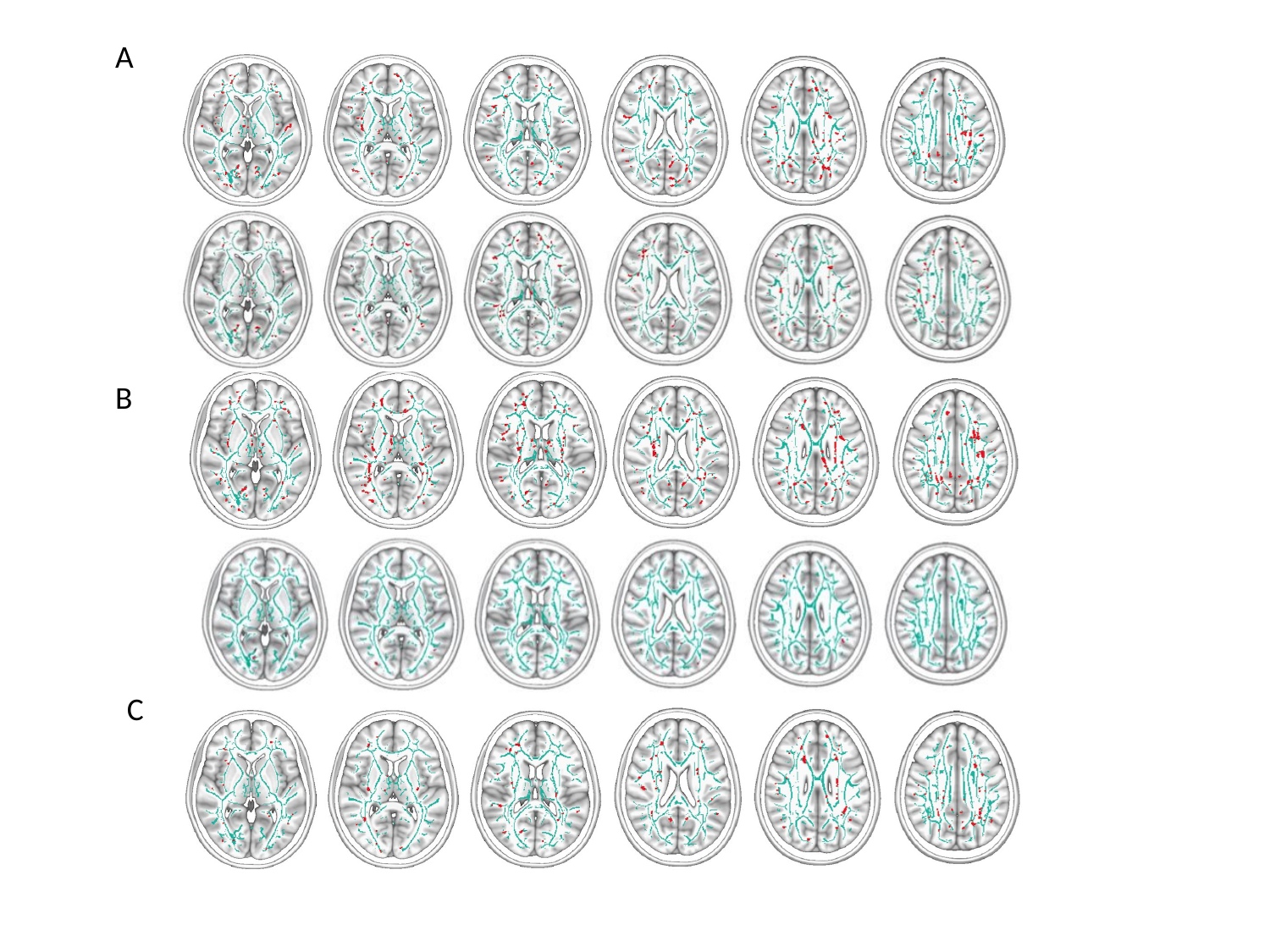

A
B
C

## Slide 5
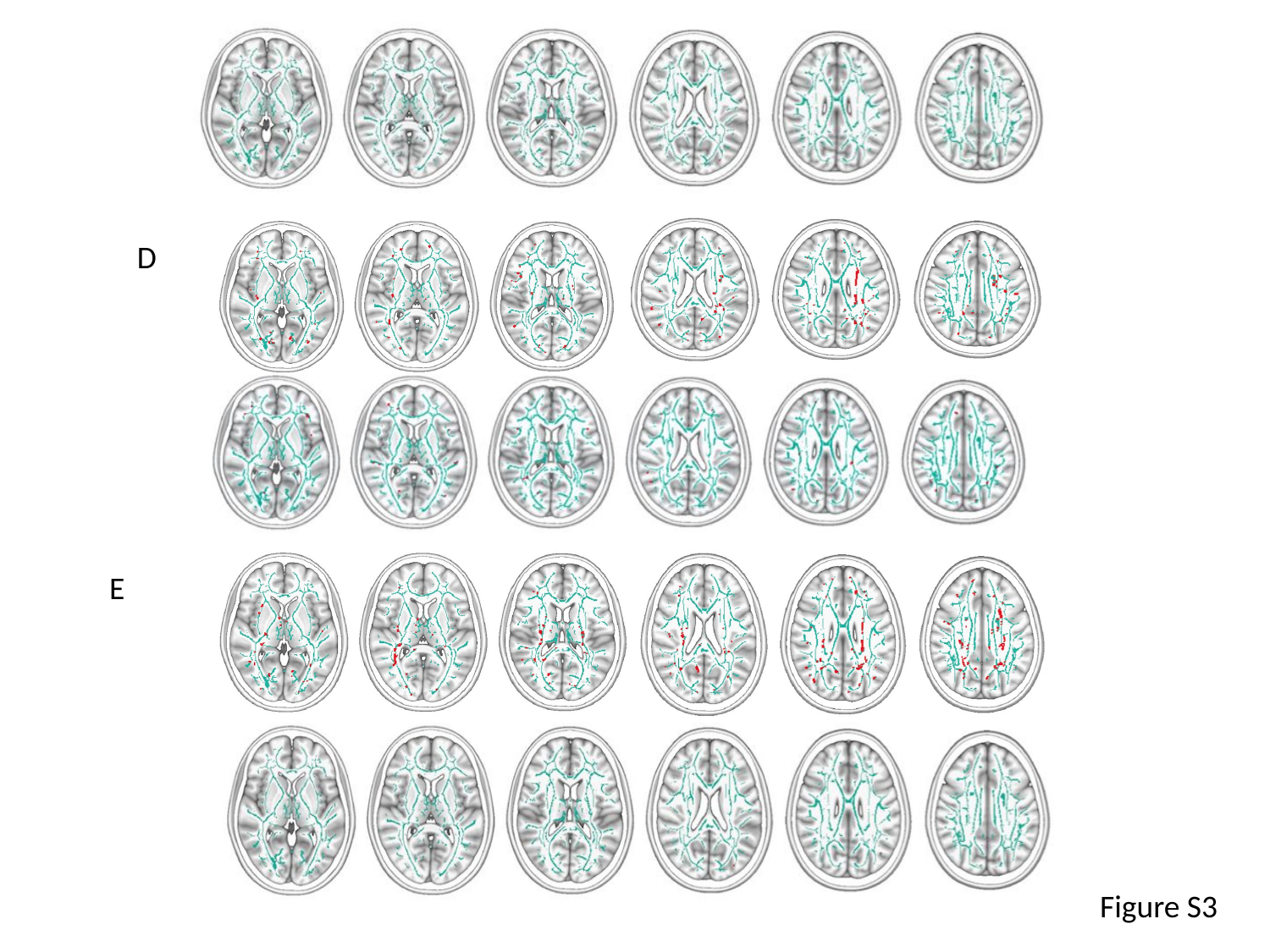

D
E
Figure S3

## Slide 6
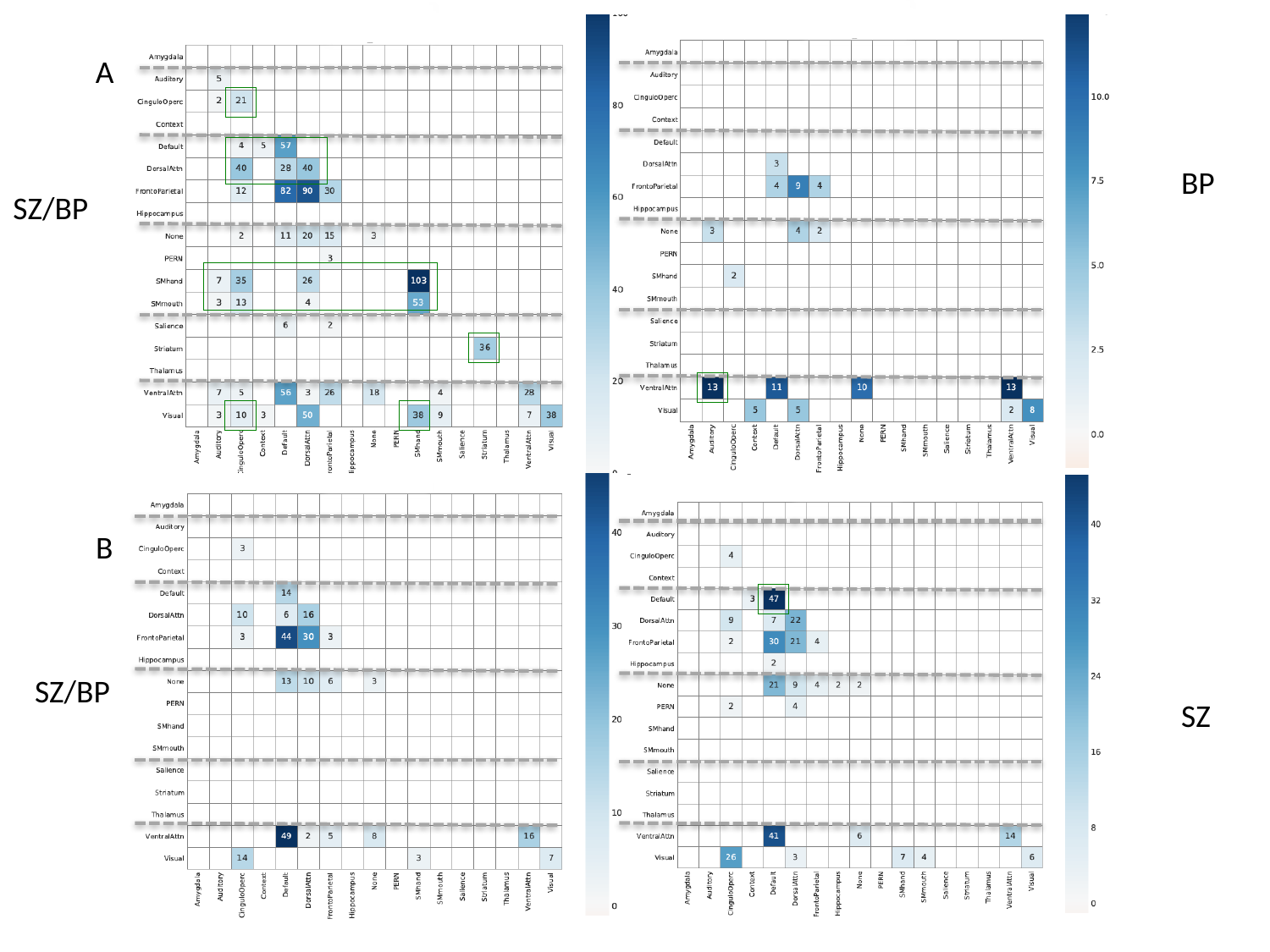

A
BP
SZ/BP
B
SZ/BP
SZ

## Slide 7
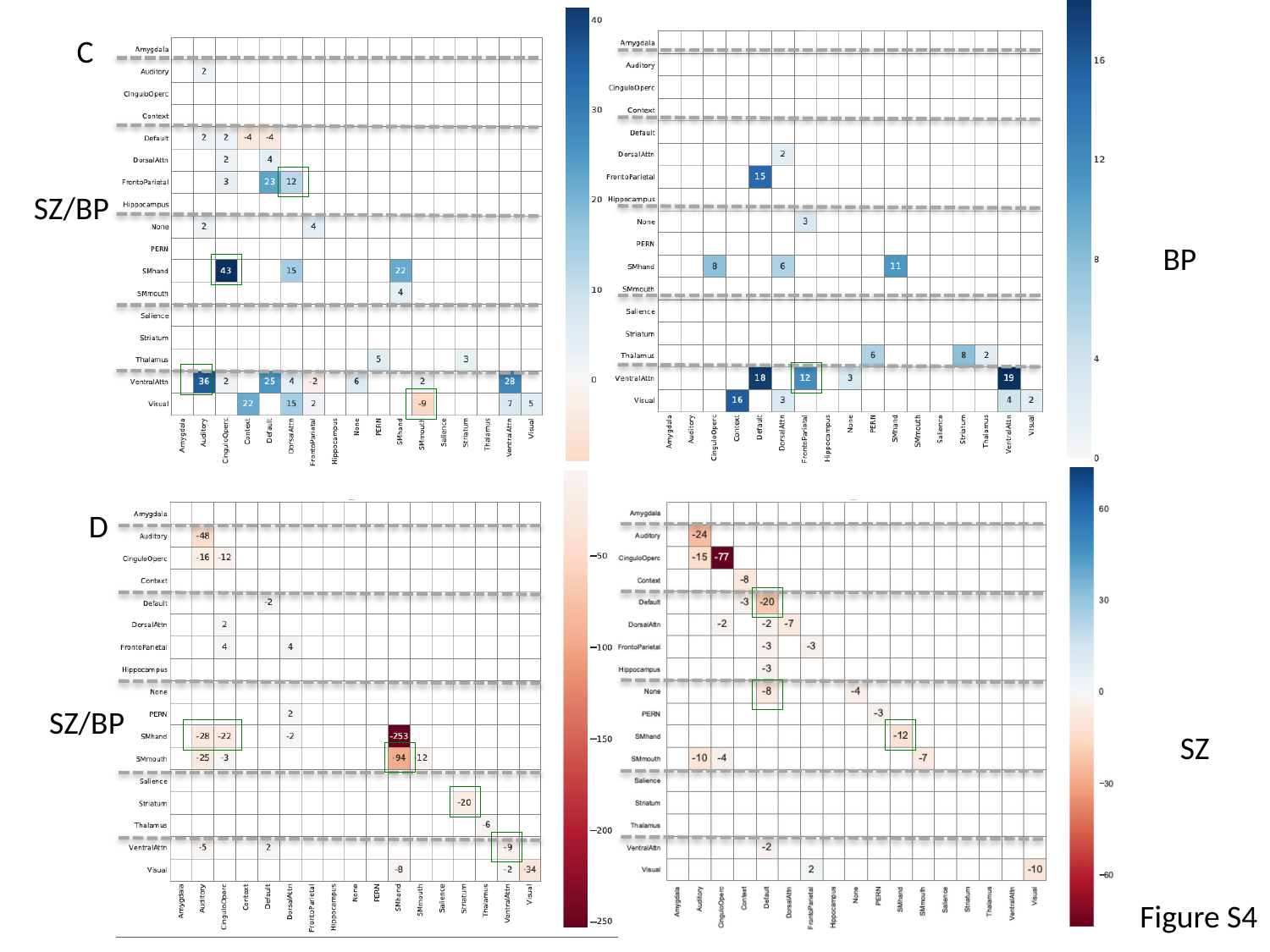

C
SZ/BP
BP
D
SZ/BP
SZ
Figure S4

## Slide 8
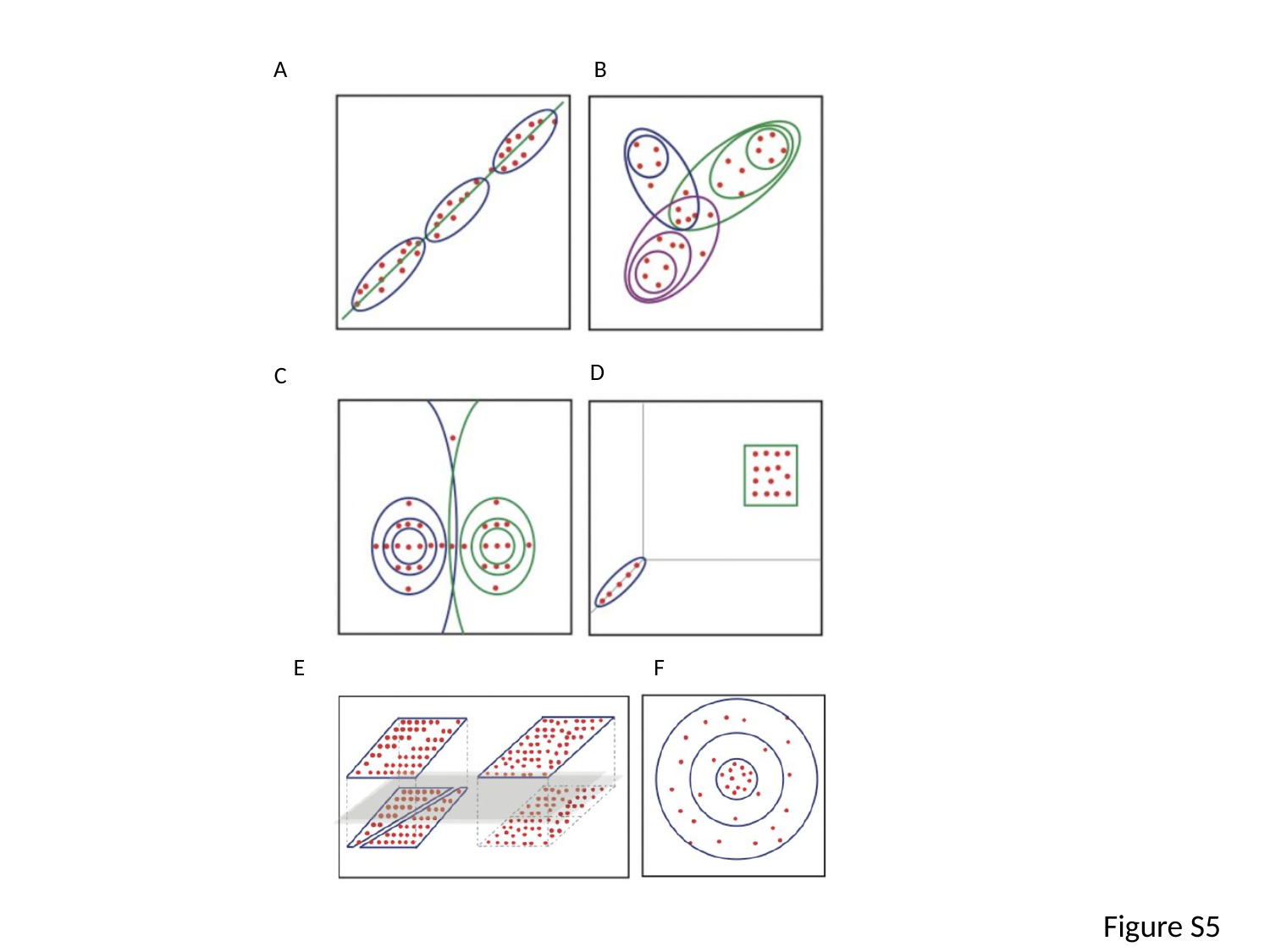

A
B
D
C
E
F
Figure S5

## Slide 9
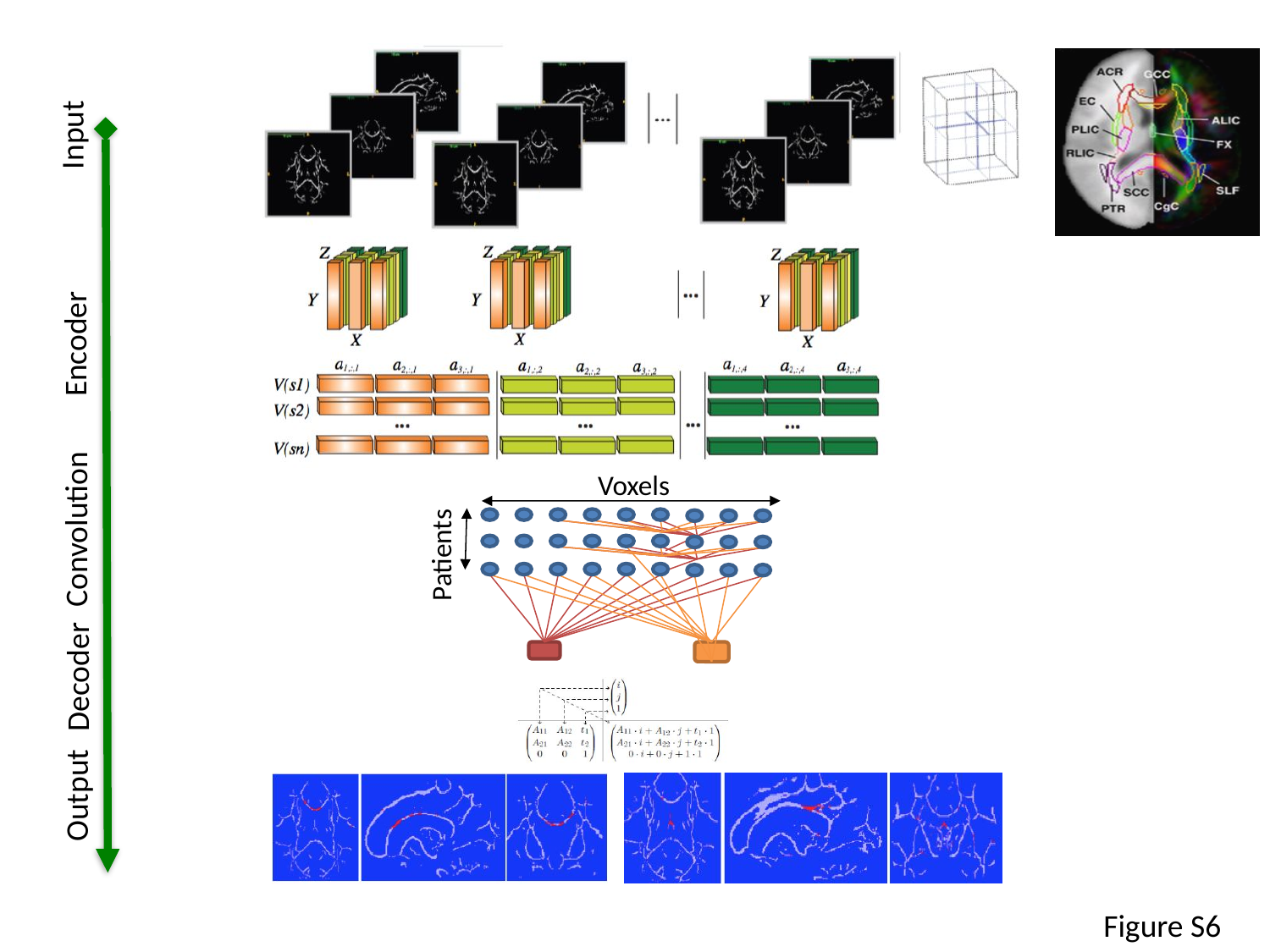

Input
Encoder
Voxels
Convolution
Patients
F’3
Decoder
Output
Figure S6

## Slide 10
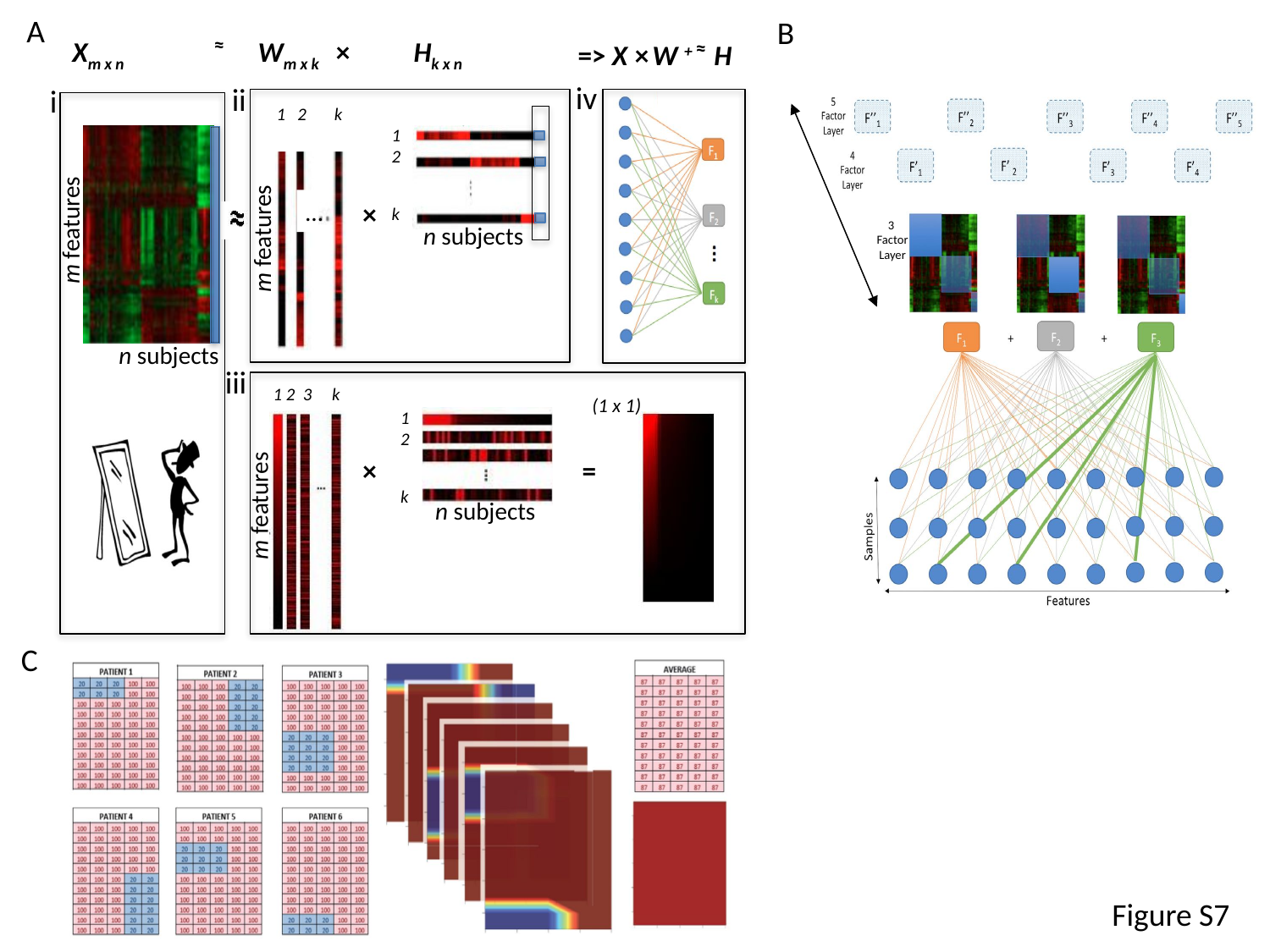

A
B
Xm x n ≈ Wm x k × Hk x n
=> X × W + ≈ H
iv
ii
i
 1 2 k
1
2
k
×
…
m features
3
Factor Layer
n subjects
m features
n subjects
iii
 1 2 3 k
(1 x 1)
1
2
k
×
=
m features
n subjects
C
Figure S7

## Slide 11
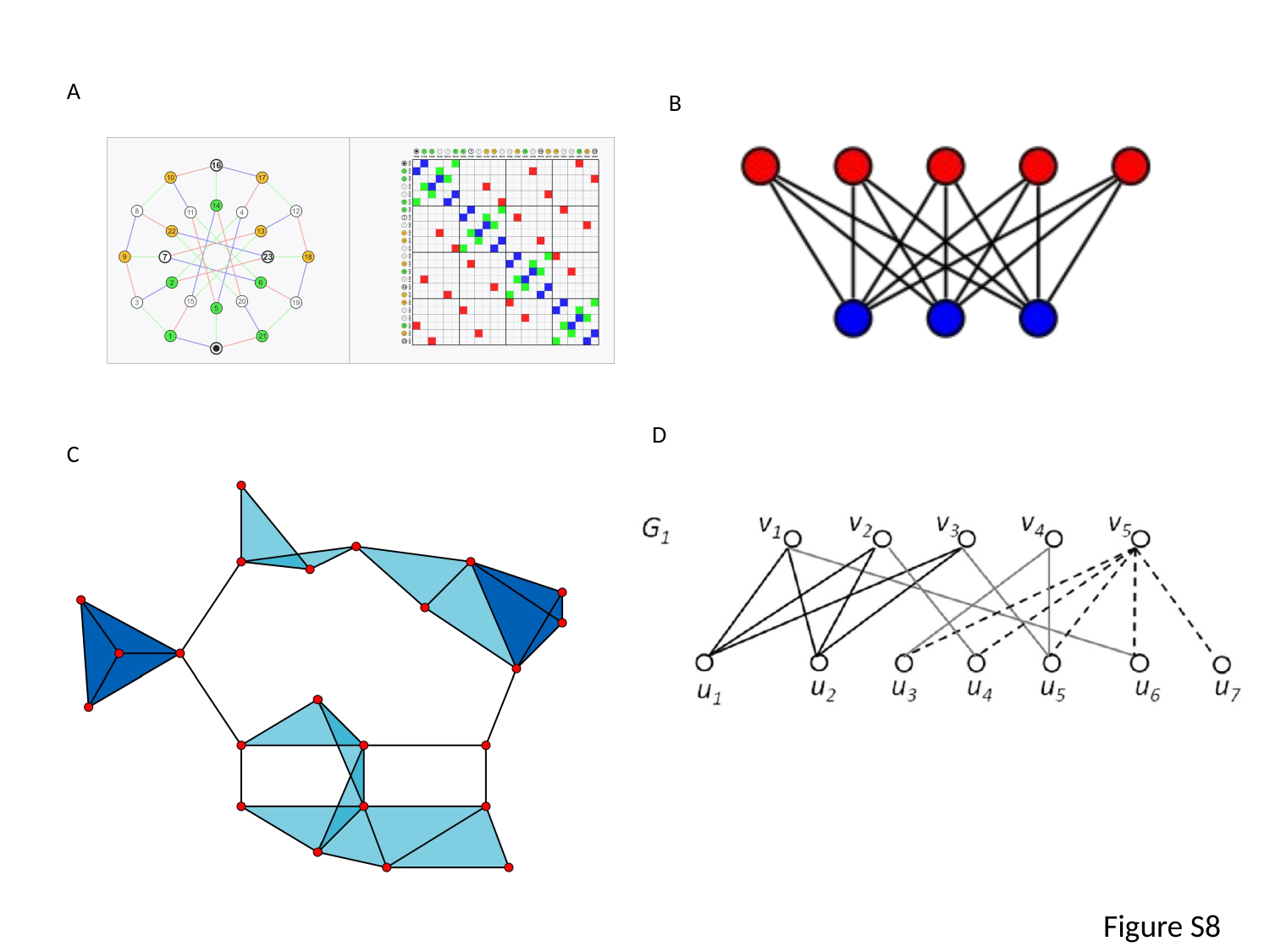

A
B
D
C
Figure S8

## Slide 12
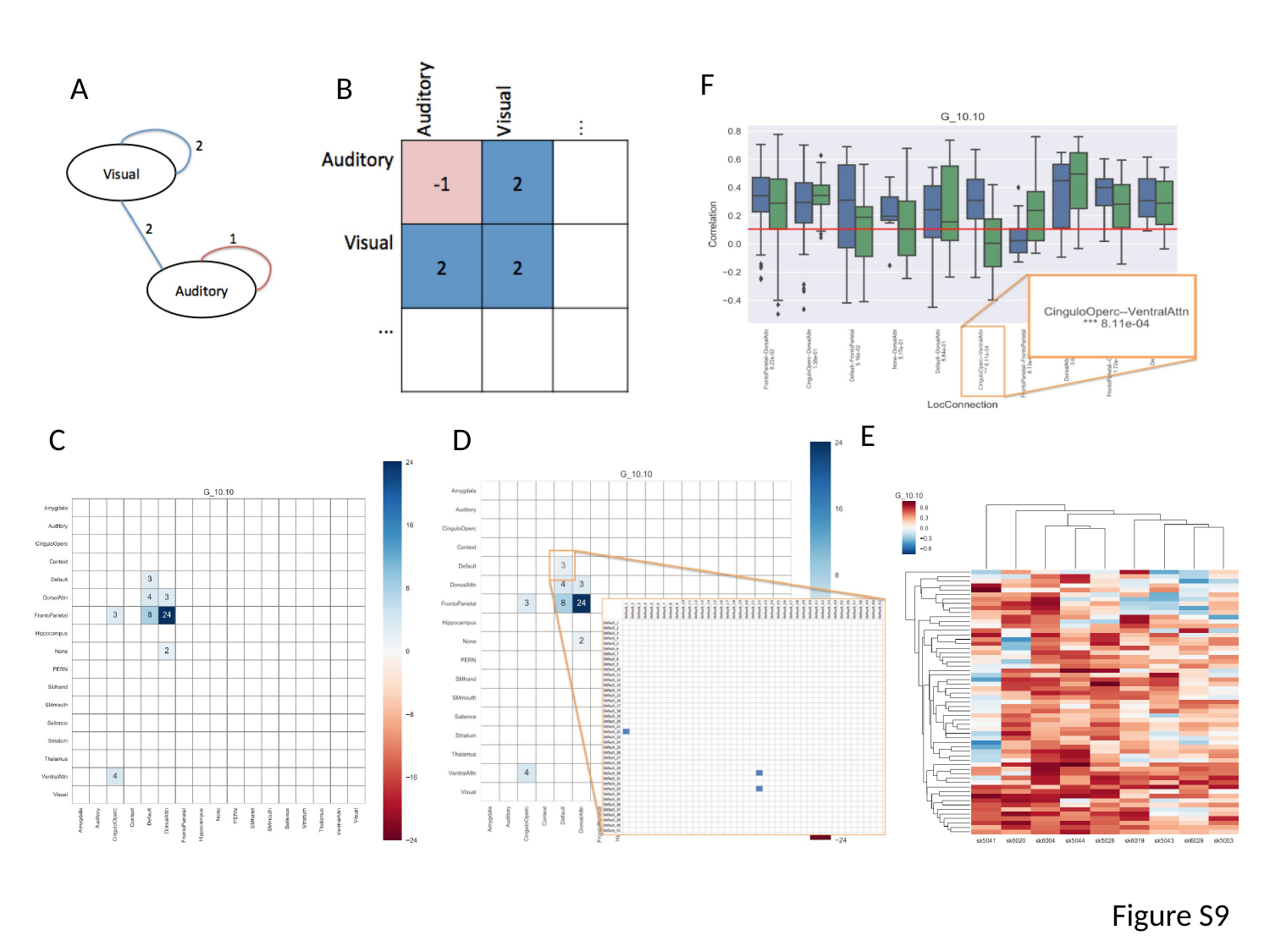

F
A
B
E
C
D
Figure S9

## Slide 13
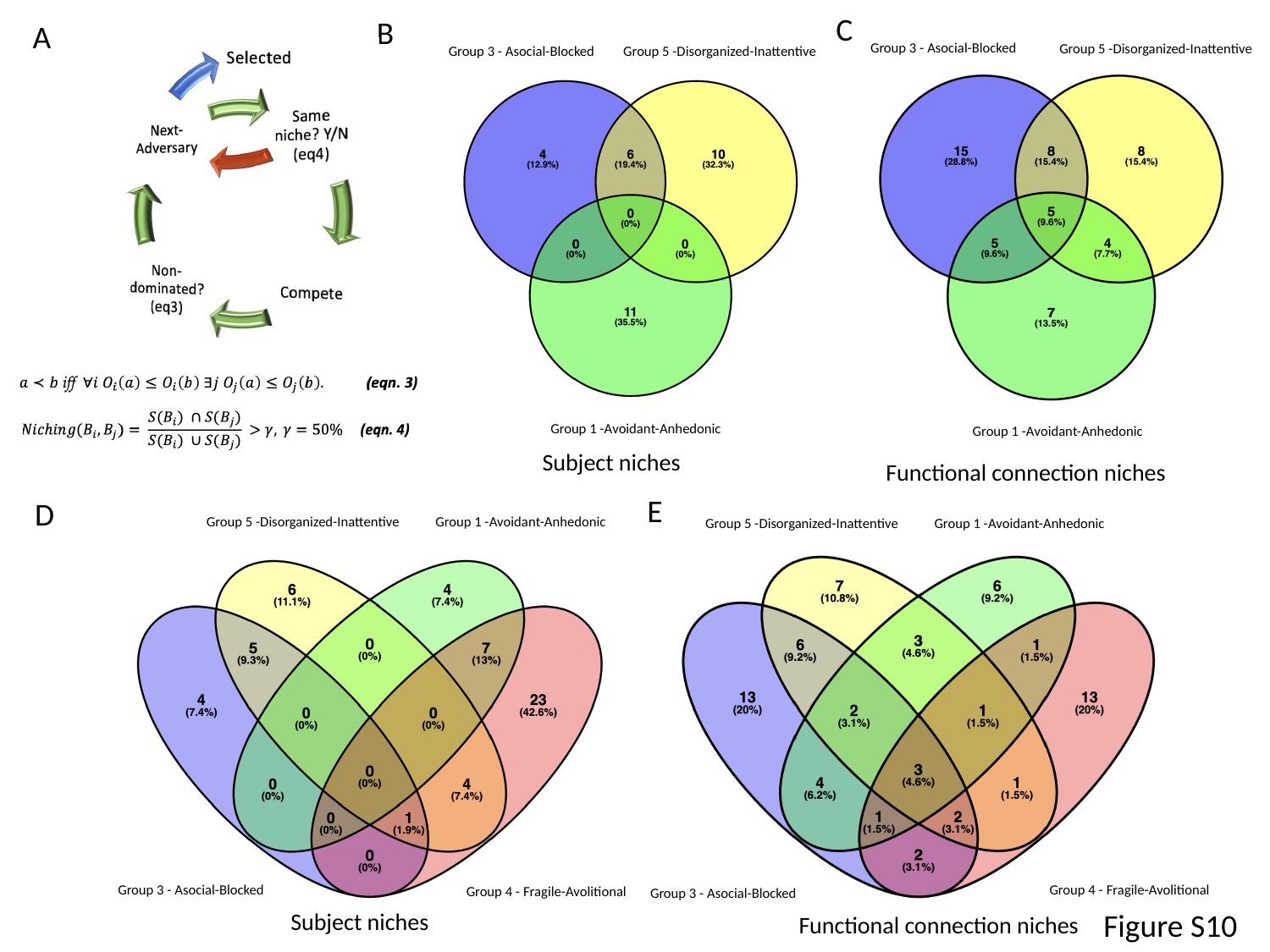

C
B
A
Group 3 - Asocial-Blocked
Group 5 -Disorganized-Inattentive
Group 5 -Disorganized-Inattentive
Group 3 - Asocial-Blocked
Group 1 -Avoidant-Anhedonic
Group 1 -Avoidant-Anhedonic
Subject niches
Functional connection niches
E
D
Group 5 -Disorganized-Inattentive
Group 1 -Avoidant-Anhedonic
Group 5 -Disorganized-Inattentive
Group 1 -Avoidant-Anhedonic
Group 3 - Asocial-Blocked
Group 4 - Fragile-Avolitional
Group 4 - Fragile-Avolitional
Group 3 - Asocial-Blocked
Figure S10
Subject niches
Functional connection niches

## Slide 14
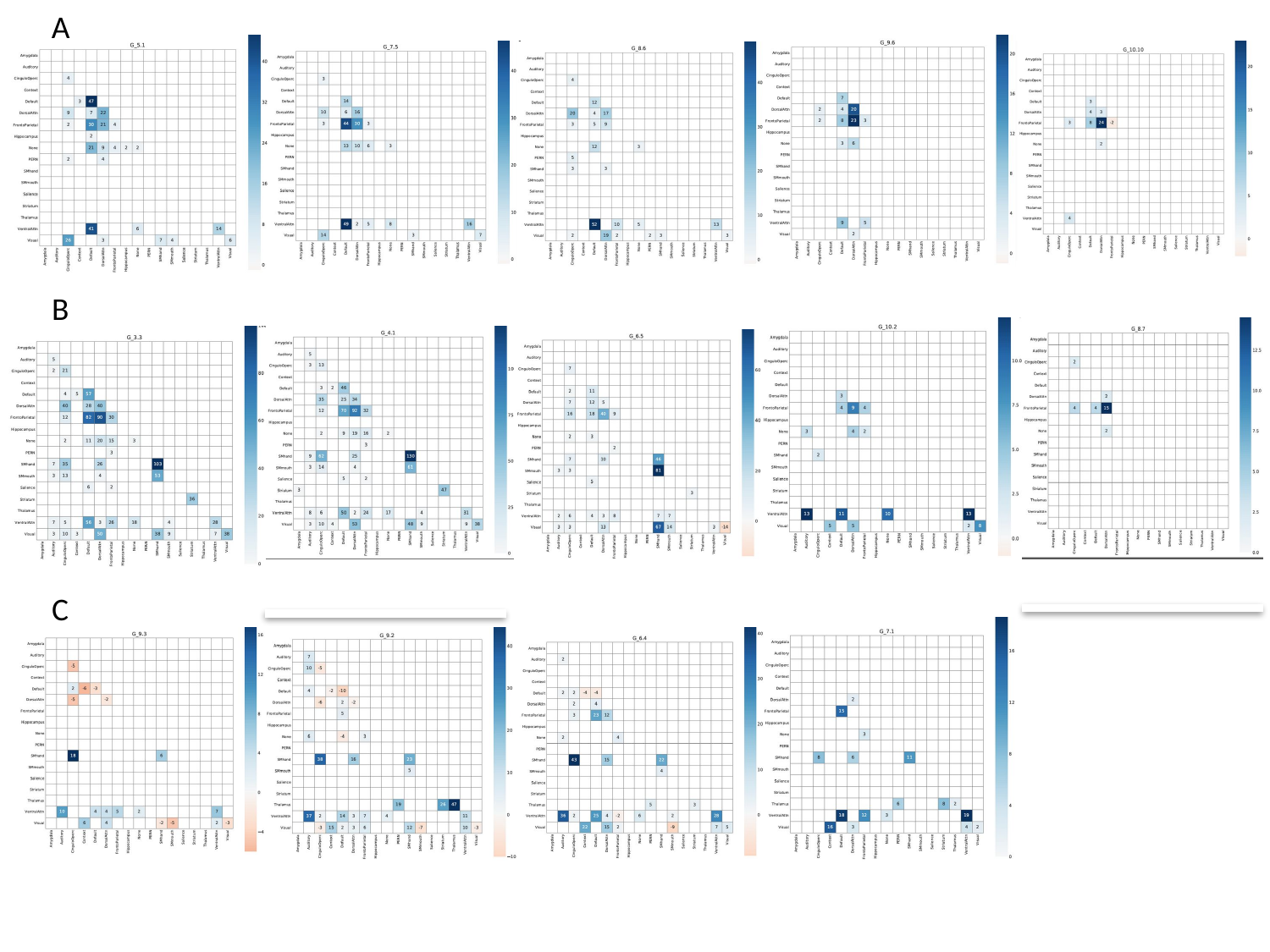

A
B
C

## Slide 15
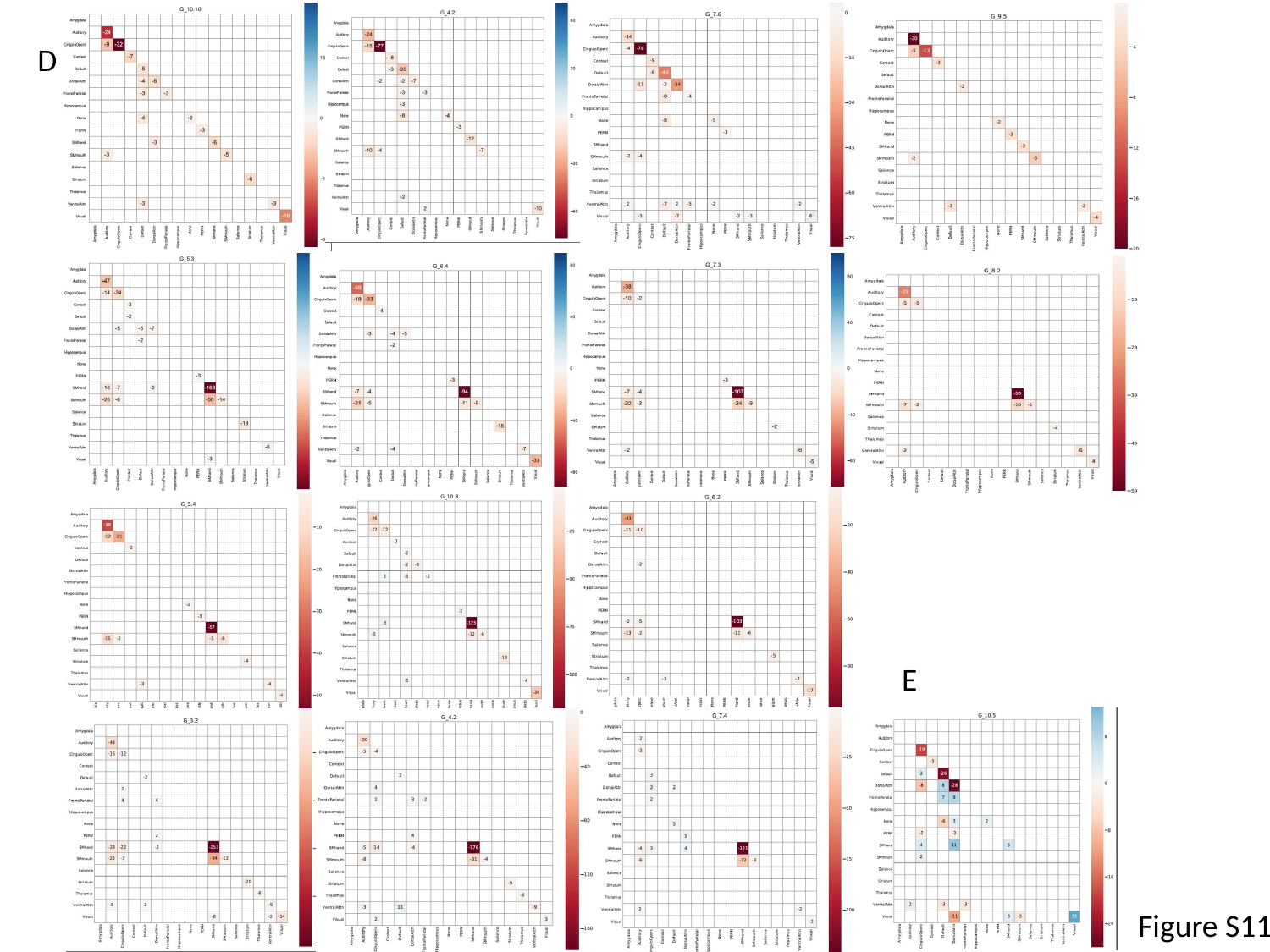

D
E
Figure S11
