# Supplementary material for: Temperament & Character account for brain functional connectivity at rest: A diathesis-stress model of functional dysregulation in psychosis
Source: Mol Psychiatry. 2023 Apr 4;28(6):2238–53. doi: 10.1038/s41380-023-02039-6 (PMC10611583; doi:10.1038/s41380-023-02039-6)
Supplement: Supplementary file 9 — Supplementary Table S7 [file 41380_2023_2039_MOESM9_ESM.docx]

**Table S7**. Comparison of demographic characteristics, duration of illness, and psychotropic medication history in uncovered rsFC groups of patients (groups 1-5) and controls. *

| Column  Variable | Row  Variable | Group  1 | Group  2 | Group  3 | Group  4 | Group  5 | Controls | Weak or no effect  % |
| --- | --- | --- | --- | --- | --- | --- | --- | --- |
|  | Number | 11 | 27 | 10 | 35 | 15 | 30 |  |
| Gender |  |  |  |  |  |  |  |  |
|  | Female | 28% | 30% | 40% | 40% | 38% | 50% | 93 |
|  | Male | 72% | 70% | 60% | 60% | 62% | 50% |  |
| Ethnicity |  |  |  |  |  |  |  |  |
|  | Black | 9% | 26% | 50% | 26% | 44% | 23% | 87 |
|  | White | 64% | 44% | 30% | 57% | 44% | 60% |  |
|  | Others | 27% | 30% | 20% | 17% | 12% | 17% |  |
| Handed-ness |  |  |  |  |  |  |  |  |
|  | Left | 0% | 11% | 10% | 0% | 12% | 10% | 93 |
|  | Right | 100% | 89% | 90% | 100% | 88% | 90% |  |
| Medication  History |  |  |  |  |  |  |  | 100 |
|  | Typical Neuro-leptic | 0% | 15% | 0% | 11% | 33% | -- |  |
|  | Atypical  Neuro-leptic | 45% | 41% | 60% | 40% | 40% | -- |  |
|  | Other mood stabilizer | 27% | 22% | 20% | 29% | 27% | -- |  |
|  | Lithium | 0% | 15% | 10% | 6% | 20% | -- |  |
|  | SSRI/SNRI | 9% | 19% | 30% | 29% | 47% | -- |  |
|  | Other anti  depressant | 18% | 7% | 0% | 17% | 7% | -- |  |
|  | BenzoDZ | 18% | 15% | 10% | 20% | 13% | -- |  |
|  | Anti-cholinergic | 0% | 0% | 10% | 9% | 0% |  |  |
| Mean Age (SD) |  | 25  (3.82) | 25  (3.20) | 25  (2.87) | 26  (3.11) | 24  (3.79) | 24  (2.99) | 100 |
| Months Illness duration  ( SD) |  | 74  (39.74) | 88  (47.16) | 81  (51.48) | 103  (59.86) | 104  (70.18) | -- | 100 |

*Notes: Handedness (right or left). Psychotropic medications include typical and atypical neuroleptics, lithium, other mood stabilizers (carbamazepine, divalproex, oxcarbazepine, topirimate, lamotrigine), SSRIs/SNRIs, other antidepressants, benzodiazepines, and anticholinergics. Duration of illness is in months. Statistical tests of inter-group differences are summarized here and detailed in Table S8. Percentage of pairwise comparisons with no significant difference (p >0.05) or weak effect size (r <0.3) is listed for each variable.
